# Is P-Glycoprotein Functionally Expressed in the Limiting Membrane of Endolysosomes? A Biochemical and Ultrastructural Study in the Rat Liver

**DOI:** 10.3390/cells11091556

**Published:** 2022-05-05

**Authors:** Birthe Gericke, Inka Wienböker, Gudrun Brandes, Wolfgang Löscher

**Affiliations:** 1 Department of Pharmacology, Toxicology, and Pharmacy, University of Veterinary Medicine, 30559 Hannover, Germany; birthe.gericke@tiho-hannover.de (B.G.); inka.wienboeker@tiho-hannover.de (I.W.); 2 Center for Systems Neuroscience, 30559 Hannover, Germany; 3 Institute of Neuroanatomy and Cell Biology, Hannover Medical School, 30625 Hannover, Germany; brandes.gudrun@mh-hannover.de

**Keywords:** hepatocytes, multidrug transport, lysosomal drug trapping, electron microscopy, early endosomes

## Abstract

The drug efflux transporter P-glycoprotein (Pgp; ABCB1) plays an important role in drug absorption, disposition, and elimination. There is an ongoing debate whether, in addition to its localization at the plasma membrane, Pgp may also be expressed at the limiting membrane of endolysosomes (ELs), mediating active EL drug sequestration. If true, this would be an important mechanism to prevent drugs from reaching their intracellular targets. However, direct evidence demonstrating the functional expression of Pgp at the limiting membrane of ELs is lacking. This prompted us to perform a biochemical and ultrastructural study on the intracellular localization of Pgp in native rat liver. For this purpose, we established an improved subcellular fractionation procedure for the enrichment of ELs and employed different biochemical and ultrastructural methods to characterize the Pgp localization and function in the enriched EL fractions. Whereas the biochemical methods seemed to indicate that Pgp is functionally expressed at EL limiting membranes, transmission electron microscopy (TEM) indicated that this only occurs rarely, if at all. Instead, Pgp was found in the limiting membrane of early endosomes and intraluminal vesicles. In additional TEM experiments, using a Pgp-overexpressing brain microvessel endothelial cell line (hCMEC/D3-*MDR1*-EGFP), we examined whether Pgp is expressed at the limiting membrane of ELs when cells are exposed to high levels of the Pgp substrate doxorubicin. Pgp was seen in early endosomes but only rarely in endolysosomes, whereas Pgp immunogold labeling was detected in large autophagosomes. In summary, our data demonstrate the importance of combining biochemical and ultrastructural methods to investigate the relationship between Pgp localization and function.

## 1. Introduction

P-glycoprotein (Pgp; MDR1; ABCB1) is a plasma membrane ATP-binding cassette (ABC) transporter, responsible for multidrug resistance in tumor cells [1,2]. In addition to tumor cells, Pgp is expressed in the plasma membrane of normal cells in many tissues, including the liver, kidney, intestine, testis, placenta, and blood–brain barrier (BBB). Pgp can protect the organism against potentially toxic xenobiotic compounds, by excreting these compounds into bile, urine, and the intestinal lumen, and by preventing their accumulation in critical organs such as the brain or testis [3,4,5,6]. In fact, Pgp is a highly versatile, if not promiscuous, drug transporter that not only affects the absorption, distribution, and elimination of toxic compounds but plays a significant role in the pharmacokinetics of a large number of therapeutically used drugs [4,7].

Localization of Pgp in the cell membrane was long thought to be the only contributor to Pgp-mediated multidrug resistance in cancer cells. In 2013, Yamagishi et al. [8] presented data supporting a novel hypothesis by which intracellular Pgp expressed in lysosomal limiting membranes of a human cervical carcinoma cell line mediates drug resistance by lysosomal drug sequestration. The authors proposed that this mechanism is enabled by the topological inversion of Pgp via endocytosis resulting in the transporter actively pumping agents into lysosomes. A similar idea was presented in 2003 by Rajagopal and Simon [9] for the ABC transporter multidrug resistance protein 1 (MRP1; ABCC1). It was long known that lysosomes can act as mediators of drug resistance by passive drug sequestration of hydrophobic weak base chemotherapeutics such as doxorubicin [10]. However, active lysosomal drug sequestration mediated by ATP-driven transporters from the ABC superfamily as reported by Yamagishi et al. [8] was thought to enable new strategies for overcoming drug resistance [10,11,12]. More recently, we suggested that active lysosomal drug trapping may also play a role in normal (non-cancer) cells by showing that lysosomal sequestration of Pgp substrates, including doxorubicin, occurs in human and porcine brain endothelial cells that form the BBB [13]. 

However, whereas the role of Pgp as a drug efflux transporter at the cell membrane is large and undisputed, one may argue that no convincing data are demonstrating that Pgp is found in lysosomal limiting membranes in normal tissues and tumors [14]. It has long been known that transmembrane proteins such as Pgp can be transported along the endocytic pathway from late endosome to lysosome, which is responsible for the degradation of the membrane proteins [15,16]. Subcellular fractionation data have suggested functionally active Pgp in lysosomal limiting membranes [8,17], but possible contamination with plasma membranes or other cell components expressing Pgp was not studied in detail. Furthermore, most studies on active lysosomal drug sequestration were performed with cancer cell lines, which may express mechanisms that are not relevant in normal tissues or tumors [14,18]. 

This prompted us to perform a study with a classical de Duve fractionation of rat liver, using the canalicular plasma membrane marker ABCB4 [19] as a negative control for the contamination of the lysosomal fraction with plasma membranes, which, to our knowledge, has not been done before in studies reporting Pgp in lysosomal membranes by subcellular fractionation. Furthermore, contamination with other organelles was examined. When using a commercial lysosome enrichment kit (also employed by Yamagishi et al. [8]) following the basic protocol provided by the manufacturer, we found that the lysosome-enriched fraction of the liver is contaminated with mitochondria, endoplasmatic reticulum (ER), and plasma membranes, which contain Pgp (reviewed by Fu [16]) and thus form a bias for any conclusions on lysosomal Pgp. Indeed, it is well known that isolation methods based on density gradient centrifugation can provide high yields of lysosomes but lysosomal fractions are often contaminated with other organelles [20,21,22,23]. Thus, in a first series of experiments, we modified the subcellular fractionation protocol to obtain an improved organelle separation. We then employed different biochemical methods to characterize the Pgp localization in the enriched lysosomal fractions. In subsequent experiments, we used different electron microscopic techniques, including immunogold labeling with lysosomal-associated membrane protein 2 (LAMP-2) and Pgp antibodies for further characterization of the localization of Pgp in the lysosome-enriched fractions. Unless otherwise stated, we will use the term endolysosome instead of lysosome, because late endosomes fuse with terminal lysosomes forming a hybrid compartment, which makes it difficult to differentiate lysosomes from late endosomes by biochemical or transmission electron microscopy (TEM) methods [24,25,26]. 

## 2. Materials and Methods

### 2.1. Animals

Female Wistar rats (250–300 g) were obtained from Charles River (Sulzfeld, Germany). Animals were kept in accordance with the guidelines of human care and use of laboratory animals of Germany with ad libitum access to food and water. All experiments were done in compliance with the German Animal Welfare Act and the European Union (EU) council directive 2010/63/EU and were formally approved by the Institutional Review Board (file # TiHo-T-2018-23). On the day of the subcellular fractionation experiment, one rat per experiment was anesthetized in a CO_2_ chamber and decapitated. The liver was removed immediately and stored on ice until washing with ice-cold phosphate-buffered saline (PBS) for further processing (Figure S1). If not mentioned otherwise, all experiments with rat liver fractions were repeated at least three times.

### 2.2. Subcellular Fractionation of Rat Liver 

Isolation of the subcellular fraction(s) from rat liver was performed using a lysosome enrichment kit (cat. #89839 Thermo Fisher Scientific, Bonn, Germany), which is identical to the kit used by Yamagishi et al. [8]. An initially used basic/standard protocol following the manufacturer’s instructions (Figure S1B) was improved (as summarized in Figure S1C) to minimize organelle contaminations and specifically characterize organelle distribution within the complete gradient after separation of the post-nuclear supernatant. Both protocols are compared in Figure S1B,C. Briefly, ~200 mg of liver tissue was rinsed three times with ice-cold PBS and mechanically homogenized in kit Solution A, containing 1% protease inhibitor (PI), using a plastic pestle on ice. Lysis efficiency was assessed under a light microscope. Next, the homogenate was mixed with kit Solution B containing 1% PI and centrifuged at 500× *g* (basic protocol) or 600× *g* (improved protocol) for 10 min at 4 °C to remove nuclei and membranous debris. The supernatant, including endosomes, endolysosomes, mitochondria, microsomes (consisting of membranes of ER, Golgi, plasmalemma, transport vesicles), and cytosol, was collected, adjusted to a concentration of 15% OptiPrep^TM^ and layered on top of a discontinuous gradient of 17%, 20%, 23%, 27%, 30% OptiPrep^TM^ (OptiPrep^TM^: 60% (*w*/*v*) solution of iodixanol in water). The OptiPrep^TM^ layer composition differed in the basic and improved protocol (see Figure S1 and Table S1). Gradient centrifugation was performed at 145,000× *g* for 2 h (basic protocol) or 8 h (improved protocol) at 4 °C in a swing-out SW 40 Ti rotor (Beckman Coulter, Brea, CA, USA). Following the basic protocol, after centrifugation, the top 2 mL of the gradient were analyzed for endolysosomal markers and contaminants whereas in the improved protocol the whole gradient was subdivided into ten 1-mL fractions that were characterized for purity and marker distribution (see Table S2). Therefore, the top 2 mL (basic protocol) or gradient fractions (improved protocol) were carefully withdrawn from the top of the gradient, mixed with ice-cold PBS, and centrifuged again at 20,000× *g* for 30 min at 4 °C to separate organelles from the cytosol. The resulting pellets were reconstituted in the appropriate buffer (supplemented with PI) dependent on the respective downstream application and either analyzed immediately or stored at −20 °C.

### 2.3. Preparation of Endolysosomal Membrane-Enriched Fractions

Based on the biochemical characterization of the subcellular fractions (F), the pellet of F1 was denoted as endolysosome-enriched fraction and was therefore used for the preparation of endolysosomal membrane-enriched fractions. Two different approaches for organelle membrane rupture were compared. In a first attempt, pelleted organelles resulting from gradient centrifugation were suspended in 50 µL fraction buffer (50 mM Tris-HCl, pH 8.0, 0.2 M NaCl, 1 mM EDTA, protease inhibitor cocktail) and subjected to three freeze–thaw (FT) cycles using dry ice/ethanol. In the second approach, fraction 1 was subjected to hypoosmotic shock (HS) in 50 µL 10 mM Tris-HCl (pH 7.4) supplemented with protease inhibitors (Complete Protease Inhibitor Cocktail, Roche, Basle, Switzerland). To separate the membrane fraction (pellet [P]) and the luminal soluble fraction (supernatant [S]) membranes were pelleted by centrifugation for 30 min at 355,000× *g* at 4 °C. The supernatant containing soluble luminal proteins was collected and stored at −20 °C. The membrane pellet was washed with 50 µL fraction buffer (FT samples) or hypoosmotic buffer (HS samples) and centrifuged at 355,000× *g* for 30 min. The remaining pellet (P) designated as the whole endolysosomal membrane fraction was solubilized in 50 µL radioimmunoprecipitation assay (RIPA) buffer containing protease inhibitors and frozen at −20 °C until Western blot analysis. 

### 2.4. Co-Immunoprecipitation

To examine a Pgp localization in LAMP-2 positive vesicles, Pgp protein levels were analyzed by Western blotting after immunoprecipitation (IP) with LAMP-2 antibody coupled beads using a crude lysosomal fraction (CLF). As described in the literature, the CLF contains endolysosomes, and a small degree of contamination with light mitochondria, peroxisomes, ER, and endosomes [27,28]. Rat liver post-nuclear supernatant (PNS) was prepared as described above and centrifuged at 20,000× *g* for 15 min to obtain a CLF. Immunoprecipitation of LAMP-2 positive structures was carried out by either coupling 50 µL protein-A sepharose (PAS) beads or 50 µL Dynabeads (1.5 mg) to LAMP-2 antibody (5 µg; cat. #PA1-655; Thermo Fisher Scientific) by preincubation for 1 h at room temperature (RT) with rotation. The bead antibody complexes were collected by centrifugation at 2000× *g* for 30 s (PAS) or using a DynaMag^TM^-2 magnetic rack (Dynabeads; Thermo Fisher Scientific) and incubated with the CLF for 2 h with constant agitation at 4 °C. The immunocomplexes were washed, eluted by boiling in 1x SDS sample buffer with dithiothreitol (DTT) for 5 min at 95 °C, and analyzed by SDS-PAGE and Western blotting.

### 2.5. Western Blotting

Western blotting was performed to biochemically assess organelle marker and Pgp distribution in all subcellular fractions. Additionally, Pgp localization in endolysosomal subcompartments and LAMP-2 immunoprecipitates was analyzed. The protein concentration in the samples was determined by the usage of Pierce BCA (*bicinchoninic acid*) Protein Assay kit (Thermo Fisher Scientific) and equal amounts of samples were loaded onto 8–10% SDS-PAGE gels. A low protein concentration in some of the gradient fractions (mainly #2 and #3) did not allow distribution analysis for all fractions of a single gradient. Thus, for LAMP-2, VDAC, Pgp, ABCB4, and calnexin distribution analysis, several gradients were quantified. Essentially, all single gradients showed a similar distribution of the different markers within the gradient. Following SDS-PAGE, proteins were transferred onto 0.45 µm polyvinylidene difluoride (PVDF) membranes. The membranes were blocked overnight at 4 °C in 5% non-fat milk (*w*/*v*) in PBS-T (PBS +0.05% Tween 20). Incubation with primary antibodies diluted in PBS-T was performed for 1 h at room temperature. Primary antibodies used and dilutions are listed in Table S3. After three washing steps with PBS-T, membranes were incubated with the appropriate secondary horseradish peroxidase coupled antibodies, either polyclonal goat anti-rabbit (1:1000; Dako, Hamburg, Germany, cat. #P0448) or polyclonal goat anti-mouse (1:1000; Dako, cat. #P0260) for 1 h at room temperature followed by another 3 washes with PBS-T. Proteins were detected by using SuperSignal West Femto Chemiluminescent Substrate (Thermo Fisher Scientific) and the Chemidoc™ XRS Imager (Bio-Rad Laboratories, Munich, Germany). Protein bands were densitometrically quantified using ImageLab version 6.0.1 (Bio-Rad, Hercules, CA, USA). The linearity of the signals and the protein concentration have been proven.

Concerning the primary antibodies for Pgp and ABCB4 used in the Western blot experiments, several different monoclonal Pgp antibodies were compared in preliminary experiments, including Sigma #P7965 (Sigma-Aldrich, Merck, Darmstadt, Germany), Thermo Fisher #MA1-26528 (=C219), Enzo #ALX-801-002 (=C219; Enzo Life Sciences, Lörrach, Germany), and Novusbio #NBP2-67667 (Novus Biologicals, Wiesbaden Nordenstadt, Germany). These antibodies recognize different extra- or intracellular epitopes of Pgp, which, however, should not be relevant for Western blot experiments. Indeed, no obvious differences in Pgp detection by Western blot were observed with these antibodies, so we chose the C219 antibody from Thermo Fisher for the main experiments shown here. This Pgp antibody, which has been developed by Victor Ling’s group [29], has been fully characterized previously and is among the most widely used Pgp antibodies [30,31]. C219, which recognizes a cytoplasmic domain of Pgp, has been validated in cells that either express or do not express Pgp. We have used C219 extensively in the past and compared it with other Pgp antibodies [4,32,33]. For the present study, we also include data obtained with the Sigma #P7965 monoclonal antibody (mAb), which recognizes an extracellular epitope of Pgp, for comparison with C219 (see Results). 

For the antibody (sc-58221 from Santa Cruz; Heidelberg, Germany) used for ABCB4 detection, we examined the specificity of this antibody by comparing Western blots of liver homogenates with those of intestinal (Caco-2), kidney (MDCKII), and brain capillary endothelial (hCMEC/D3) cell lines that do not express ABCB4 [19,34]. This was done by protein separation using 4–20% Mini-PROTEAN gels (Bio-Rad), immunoblotting and normalization to total protein per lane by Stain-Free technology. Caco-2 cells were obtained from DSMZ (Brunswick, Germany), MDCKII cells were kindly provided by Prof. Piet Borst (The Netherlands Cancer Institute, Amsterdam, Netherlands), hCMEC/D3 cells by Prof. Pierre-Olivier Couraud (INSERM U 1016, Institut Cochin, Paris, France). As shown in Figure S2, the results confirm the specificity of this antibody previously reported by Stremmel et al. [34]. 

### 2.6. Assessment of Gradient Fractionation Efficiency and Distribution of Pgp in the Marker Organelle Enriched Fractions

Lysosomal yield or recovery after gradient centrifugation and enrichment within the gradient fractions was calculated based on Western blot analysis of the endolysosomal marker LAMP-2. Likewise, recovery and enrichment were calculated for Pgp. The following formulas were used.


$$\mathrm{Recovery}\;\left(\%\right)=\frac{\left({\mathrm{band}\;\mathrm{intensity}}_{LAMP2\;or\;Pgp}/µg\;\mathrm{of}\;\mathrm{total}\;\mathrm{protein}\;\times \;\mathrm{total}\;\mathrm{protein}\;\mathrm{amount}/\mathrm{fraction}\right)}{\left({\mathrm{band}\;\mathrm{intensity}}_{LAMP2\;or\;Pgp}/µg\;\mathrm{of}\;\mathrm{total}\;\mathrm{protein}\;\times \;\mathrm{total}\;\mathrm{protein}\;\mathrm{amount}\;\mathrm{in}\;\mathrm{liver}\;\mathrm{homogenate}\right)}$$

$$\mathrm{Fold}\;\mathrm{enrichment}=\frac{{\mathrm{band}\;\mathrm{intensity}}_{LAMP2\;or\;Pgp\;}\mathrm{fraction}}{{\mathrm{band}\;\mathrm{intensity}}_{LAMP2\;or\;Pgp}\;\mathrm{liver}\;\mathrm{homogenate}}$$

We also performed these calculations based on PNS but data were similar to those based on liver homogenates as a comparator, so only the latter data are reported here. 

### 2.7. Acid Phosphatase (AP) Assay

The distribution of the endolysosomal hydrolase acid phosphatase (AP) in the gradient fractions was determined using an AP assay kit (Sigma-Aldrich, cat. #CS0740) following the manufacturer’s instructions. Briefly, organelles from each gradient fraction were pelleted as described above. Pellets were solved in 0.09 M citrate buffer containing 0.5% Triton X-100 for the release of endolysosomal enzymes. After permeabilization of endolysosomal membranes by the detergent for 15 min at room temperature, triplicates of each fraction were added to a 96-well microtiter plate and mixed with substrate solution (4-nitrophenyl-phosphate in 0.09 M citrate buffer, pH 4.8). The plate was incubated for 30 min at 37 °C to allow enzymatic conversion of 4-nitrophenyl-phosphate into p-nitrophenol. To stop the reaction, 0.5 M NaOH was added to the wells resulting in p-nitrophenolate with an intense yellow color under alkaline conditions. Absorption was measured at a wavelength of 405 nm using a Dynatech MRX microplate reader (Dynatech, Guernsey, Great Britain). Protein concentrations in the fractions were determined by the BCA assay. The AP activity was expressed in units per mL.

### 2.8. Rhodamine 123 Uptake Assay Using the Crude Lysosomal Fraction (CLF) of Rat Liver

Functionality, i.e., active transport of substances by the action of Pgp, located within the late endocytic vesicle membrane, was evaluated by uptake of the cell-permeant, cationic, green-fluorescent Pgp substrate rhodamine 123 (Rho123) into the vesicular lumen in the presence or absence of the Pgp specific inhibitor tariquidar. The assay was performed using a CLF prepared from fresh rat liver as described above. The CLF was either pre-incubated with 0.5 µM tariquidar (control samples) or vehicle (DMSO) in OptiMEM without phenol red for 1 h (37 °C, 5% CO_2_) and agitation at 55 rpm. After an additional PBS wash, samples were incubated with 2 µM of the lipophilic green fluorescent Pgp substrate Rho123 (2 µM) either in the presence (control samples) or absence of 0.5 µM tariquidar for 120 min (37 °C, 5% CO_2_) with agitation at 55 rpm. After another wash step of the CLF with PBS and centrifugation at 20,000× *g* for 15 min, the vesicle pellet was lysed in 150 µL RIPA buffer. Membranous debris was removed by centrifugation at 10,000× *g* for 10 min and absolute Rho123 fluorescence in the supernatant was measured with a FLUOstar OPTIMA (BMG Labtech, Ortenberg, Germany) plate reader. The experiment was repeated four times and samples were run in triplicates.

### 2.9. Electron Microscopy of the Subcellular Fractions and Immunopurified Vesicles

To evaluate the purity of the gradient fractions, F1–F4 and F8 were adsorbed to a Formvar-coated copper grid (Plano GmbH, Wetzlar, Germany) covered by a 20 nm thick carbon layer, incubated with 3% uranyl acetate (Serva, Heidelberg, Germany) for 30 s followed by washing with aqua dest and drying. 

Additionally, a pull-down assay was performed with gradient fractions F1–F4 and, as a negative control, F8 to compare the morphology of LAMP-2 and Pgp-positive vesicles. Therefore, Dynabeads (Thermo Fisher Scientific #10007D, 1.5 mg) were coupled either to an antibody against LAMP-2 (Thermo Fisher Scientific; cat. #PA1-655, 1:500) or Pgp (Enzo; cat. #ALX-801-002, 1:500) by preincubating for 30 min at room temperature with rotation. Both antibodies specifically recognize cytoplasmic epitopes of the respective protein. Pelleted fractions 1–4 and 8 obtained by density gradient centrifugation were suspended in 1 mL PBS containing PI. A volume of 500 µL of each fraction was incubated with either anti-LAMP-2 or anti-Pgp coupled Dynabeads overnight at 4 °C on a rotating mixer. The resulting immunocomplexes bound to the Dynabeads were washed three times with the supplied washing buffer and solubilized in 20 µL 0.1 M sodium cacodylate, pH 7.3 supplemented with PI.

A total volume of 15 µL from each sample was adsorbed on carbon-coated films stabilized by a copper grid and stained with 3% uranyl acetate. In addition, the remaining immunoprecipitates were pelleted, fixed by 2.5% glutaraldehyde (Polysciences, Warrington, PA, USA) in 0.1 M sodium cacodylate (Merck, Darmstadt, Germany), pH 7.3 and postfixed in 2% osmium tetroxide (Polysciences) in 0.1 M sodium cacodylate, pH 7.3. After dehydration in graded alcohols (J.T.Baker, Phillipsburg, NJ, USA) and incubation in Toluol (Merck), the specimens were embedded in epoxide resin (Serva). Ultrathin sections were stained with 2% uranyl acetate and lead citrate (Serva).

All specimens were examined under the transmission electron microscope (TEM; Morgagni 268, 80 kV, FEI, Eindhoven, The Netherlands). The digital images were processed using Adobe Photoshop CS6 (Adobe, San José, CA, USA).

### 2.10. Ultrastructure of the Rat Liver

The anesthetized rat was perfused with 0.01 M PBS followed by the fixative 4% paraformaldehyde in PBS. The liver was extracted and cut into small pieces. The tissue pieces were incubated in 1% Tris-buffered saline for 30 min and cells were permeabilized with 0.1% Triton-X-100 in PBS. The first incubation with the antibodies anti-LAMP-2 (Thermo Fisher Scientific; cat. #PA1-655, 1:500) and anti-Pgp (Thermo Fisher Scientific; cat. #MA1-26528, 1:500; Enzo; cat. #ALX-801-002, 1:50), recognizing cytoplasmic epitopes, respectively, diluted in 0.01% Triton-X-100 and 3% bovine serum albumin in PBS was carried out at 4 °C overnight. After rinsing in the same buffer, the second gold-labeled antibodies goat anti-rabbit-IgG H&L (20 nm gold particles; Abcam, Cambridge, UK) or anti-mouse-IgG H&L (10 nm gold particle; Abcam) were incubated at a dilution of 1:500 in the same buffer for 1 h at room temperature. Following another rinsing step, the specimens were fixed in 2% glutaraldehyde in 0.1 M sodium cacodylate, pH 7.3, and were postfixed in 2% osmium tetroxide in the same buffer. After dehydration in graded alcohols and embedding in epoxide resin, only the surface of the specimens was sectioned. Ultrathin sections were stained with 2% uranyl acetate and lead citrate. After examination under the TEM, the digital images were processed using Adobe Photoshop CS6.

Concerning the primary antibodies for Pgp used in the immune-TEM experiments described in 2.9 and 2.10, several different Pgp mAbs were compared in preliminary experiments, including Sigma #P7965, Santa Cruz #sc-8313 (H-241), Thermo Fisher #MA1-26528 (=C219), Enzo #ALX-801-002 (=C219), and Novusbio #NBP2-67667. As described in 2.5, these antibodies recognize different extra- or intracellular epitopes of Pgp, which may be relevant for immune TEM experiments. Indeed, the Sigma antibody #P7965, which recognizes an extracellular epitope of Pgp, was particularly suited to detect Pgp at the plasma membrane, whereas antibodies recognizing intracellular Pgp epitopes were better suited to detect Pgp in intracellular organelles such as endosomes and endolysosomes, which could be due to the topological inversion of Pgp in these organelles described in the Introduction. Only the sc-8313 antibody from Santa Cruz did not exhibit specific binding. Thus, given the aims of the present study, we chose the C219 antibody from Enzo for the main experiments shown here.

### 2.11. Electron Microscopy of hCMEC/D3 Cells with vs. without Exposure to Doxorubicin

To collect TEM evidence that serves as proof or disproof that Pgp would be expressed at the limiting membrane of endolysosomes in cells exposed to high levels of Pgp substrates, we used a human brain capillary endothelial cell (BCEC) line (hCMEC/D3). As reported previously [35,36,37], we transduced these cells with a doxycycline-inducible *MDR1*-EGFP fusion plasmid [35]. In the presence of doxycycline, these cells exhibit a 15-fold increase in Pgp-EGFP fusion protein expression, which is associated with an increased efflux of the Pgp substrate Rho123 [36]. 

For the present experiments, the hCMEC/D3-*MDR1*-EGFP cell line was cultivated as previously described [13]. Pgp-EGFP protein expression was induced by cell cultivation in a medium containing 1 µg/µL doxycycline. Before TEM, hCMEC/D3-*MDR1*-EGFP cells were exposed to the Pgp substrate doxorubicin (10 µM) for 30 min at 37 °C. Sham exposed cells were used for comparison. Immune-TEM of the cells was performed as described in Section 2.10 above. For Pgp, the Sigma #P7965 mAb, which recognizes an extracellular epitope of Pgp, was used in a dilution of 1:500, whereas the anti-LAMP-2 antibody was the same as used for the liver preparations. 

### 2.12. Statistics

Depending on whether data were normally distributed or not, parametric or nonparametric tests were used for statistical evaluation. For comparison of two independent groups, Student’s t-test or the Mann–Whitney U-test were used. All statistical analyses were performed with the Prism 8 software from GraphPad (La Jolla, CA, USA). Two-sided tests were used; a *p* < 0.05 was considered significant.

## 3. Results

To study a potential function of Pgp in the endocytic system, the localization of Pgp in endolysosome-enriched subcellular fractions of rat liver was analyzed by combining biochemical and morphological methods. The endolysosomal enrichment from rat liver tissue was performed by combining low-speed centrifugation for clearance of rat liver homogenate from nuclei and cell debris and separation of the resultant PNS by density gradient centrifugation (Figure S1). Organelle separation, endolysosomal enrichment, and purity were biochemically characterized by analyzing the presence of marker proteins for various organelles (Table S2) within the gradient fractions using Western blotting.

### 3.1. Biochemical Characterization of the Endolysosome-Enriched Fraction Obtained by Using the Basic Fractionation Protocol

Using the lysosome enrichment kit of Thermo Fisher/Pierce according to the vendor’s instructions (see summary in Figure S1B), the endolysosomal marker protein LAMP-2 was enriched in the top 2 mL of the gradient as expected and co-distributed with Pgp in the same fraction (Figure S3). However, the endolysosome-enriched fraction also contained a low amount of ABCB4 (a canalicular plasma membrane marker). Thus, it cannot be excluded that at least part of the Pgp in the endolysosome-enriched fraction stems from contamination with plasma membranes. Furthermore, the endolysosome-enriched fraction contained calnexin (an ER marker), and VDAC (a mitochondrial marker), which would indicate additional contamination with organelles that may contain Pgp. Thus, we decided to improve the initial gradient fractionation protocol to obtain optimized isolation of endolysosomes.

### 3.2. Biochemical Analysis of Pgp and Organelle Marker Distribution in Subcellular Fractions following an Improved Fractionation Protocol

**Figure 1:**
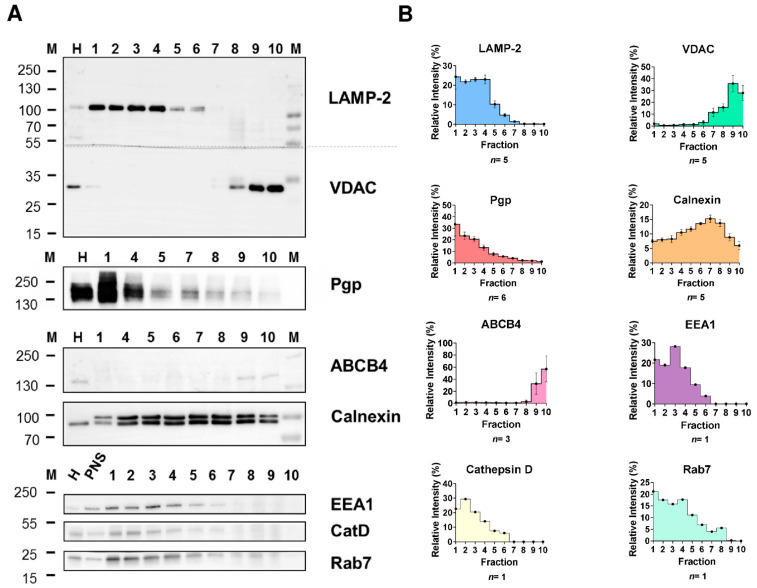
Biochemical characterization of rat liver subcellular fractions and Pgp localization in endolysosomal-enriched fractions. Rat liver subcellular fractionation was performed by low speed centrifugation and subsequent density gradient ultracentrifugation. Recovery and purity of the cell organelles from collected gradient fractions 1–10 (top to bottom) were analyzed by separation of equal protein amounts (5 µg) of each fraction and whole-cell homogenate (H) and post-nuclear supernatant (PNS) by SDS-PAGE and immunoblotting. Homogenate and PNS data were similar; thus, PNS is shown here for only some proteins. Endolysosomes were detected using the LAMP-2 and Rab7 marker proteins and the luminal lysosomal protease cathepsin D (CatD). EEA1 was used as a marker for early endosomes. The purity of the endolysosomal fractions was assessed by organelle markers for mitochondria (VDAC), canalicular plasma membrane (ABCB4), and ER (calnexin). (**A**) Representative Western blots showing the distribution of the different organelle protein markers and Pgp in the gradient fractions. Some fractions (mainly 2 and 3) contained low protein amounts and thus could not be characterized for the presence of all marker proteins for each individual gradient. Therefore, marker quantification in the gradient fractions was repeatedly performed for different fractionations. (**B**) Quantification of the protein band intensities for the marker proteins in the different gradient fractions. Immunoblotting revealed recovery of endolysosomal vesicles from the top of the gradient (fractions 1–4) and co-fractionation of Pgp in the enriched endolysosomal fractions (1–4). Fraction 1 was essentially free of canalicular plasma membrane contamination and mitochondria but contains remnants of ER. Mitochondria localized in fractions 7–10 and were clearly separated from endolysosomal markers. The ER marker calnexin was present in each fraction, with the lowest amount in endolysosomal fraction 1. Values are expressed as the percentage of total marker protein levels detected in all fractions combined (set to 100%). Data are presented as means ± SEM; the number of quantified Western blots (*n*) is shown below the graphs.

To improve gradient organelle separation, the subcellular fractionation protocol was optimized as shown in Figure S1C and Table S1. After centrifugation, the gradient was subdivided into 1 mL fractions denoted as F1–F10 (from top to bottom) and analyzed for organelle distribution by Western blotting. As shown in Figure 1, LAMP-2 and Pgp were enriched in gradient F1–F4 and clearly separated from mitochondrial contamination as visible by mitochondrial marker (VDAC) distribution to F6–F10 or F7–F10, depending on individual gradients. The presence of high levels of Pgp in the LAMP-2 enriched fractions (F1–4) detected by the C219 Pgp mAb was confirmed by another mAb that recognizes an extracellular epitope of Pgp (Figure S4). 

In comparison to enriched endolysosomal organelles obtained using the initial gradient fractionation protocol, the mitochondrial marker VDAC was entirely absent in the LAMP-2 enriched fractions. Thus, our optimized isolation procedure effectively separated endolysosomal vesicles from mitochondria. Furthermore, again in contrast to the initial gradient fractionation protocol, fractions 1–4 were essentially free of the canalicular plasma membrane marker ABCB4. However, contamination with the ER marker calnexin was detected. Concerning ABCB4, it is important to note that, in contrast to other canalicular membrane transporters such as ABCB11, ABCB4 hardly detectable in the endocytic system of hepatocytes [38], which could explain that ABCB4 was not detected in fractions corresponding to intracellular pools (Figure 1). Expression of ABCB4 is largely restricted to the canalicular membrane of hepatocytes [19], which was also shown here by demonstrating that the ABCB4 mAb detected the transporter in liver homogenates but not in intestinal and renal cells or BCECs (Figure S2).

In addition to LAMP-2, two other late endolysosomal markers (cathepsin D [CatD] and Rab7) and the early endosomal marker EEA1 were concentrated in fractions F1–F4 (Figure 1). Therefore, F1–F4 were denoted as endocytic organelle enriched gradient fractions. 

Taken together, our isolation procedure led to the enrichment and purification of endocytic vesicles. To further characterize endolysosome-enriched fractions and validate endolysosomal localization and enrichment after gradient centrifugation, the activity of the luminal endolysosomal enzyme AP was measured in F1–F10. AP is considered specific for the late endolysosomal system [25,39,40]. Thus, the detection of enzyme activity after solubilization of the membrane is a measure of the presence and structural integrity of the gradient-enriched endolysosomal vesicles. However, in rat liver, AP has also been determined in early endosomes [41], most likely because the enzyme is transported, at least in part, via a constitutive pathway from Golgi via the cell membrane to endosomes and endolysosomes [42,43]. 

**Figure 2:**
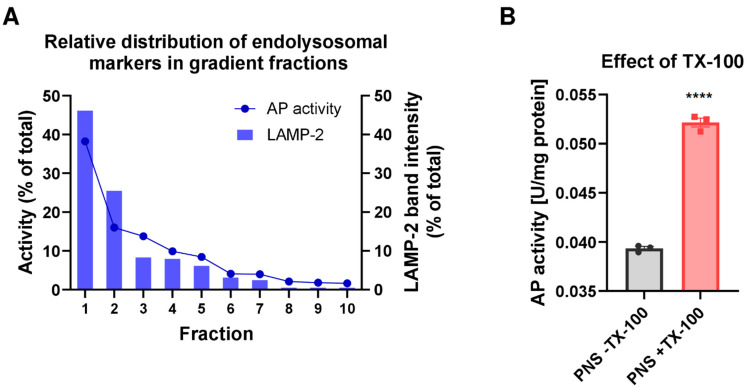
The activity of the luminal endolysosomal enzyme acid phosphatase in the gradient fractions in comparison to LAMP-2 distribution. (**A**) The activity of the luminal endolysosomal enzyme acid phosphatase (AP) was assessed in density gradient fractions to further define endolysosome-enriched fractions. AP activity in the gradient fractions was measured after endolysosomal membrane lysis with Triton X-100 (TX-100) and calculated in units (U) per mL. For each fraction, the percentage of total activity in all fractions combined (set to 100%) is plotted in comparison to the percentage of total recovered LAMP-2 in all fractions. The total LAMP-2 amount in each fraction was obtained by calculating the product of band intensity/µg total protein and total protein amount in the fraction. A peak for the relative AP activity as well as the endolysosomal marker protein LAMP-2 was detected in F1, indicating that the bulk of endolysosomes was contained in this fraction. Data were collected from one representative gradient. (**B**) Diagram showing the effect of TX-100 treatment on measured AP activity. The increase in AP activity after the addition of TX-100 to the post-nuclear supernatant (PNS) reflects the efficiency of organelle lysis and the structural integrity of organelles present in the PNS loaded onto the gradient. AP activity is presented in U/mg of protein. Data are shown as individual values and the median of three replicates. The significance of the difference is indicated by asterisks (**** *p* < 0.00001).

As shown in Figure 2, the highest activity of AP was determined in the first gradient fraction F1. The activity progressively decreased in the lower gradient fractions and was nearly absent in the last gradient fractions. These results were in line with the LAMP-2 protein distribution as examined by Western blotting when data were shown in percent of total LAMP-2 (Figure 2A). As AP activity and LAMP-2 levels depicted in Figure 2A are shown in relation to the fraction volume, results indicate that the highest absolute amount of the endolysosomes was present in the first fraction (F1). This observation is also consistent with the high protein amount recovered in the first gradient fraction (Figure S5). Indeed, our subcellular fractionation procedure yielded a recovery of up to 35% of the endolysosomal vesicles present in the initial liver homogenate, as determined by densitometric quantification of LAMP-2 levels in gradient fraction 1 (F1). Furthermore, the fractionation resulted in an approximately 10-fold enrichment of LAMP-2 compared to liver homogenate in the first gradient fraction (Table S4). Based on LAMP-2 recovery, the total endolysosomal protein in the rat liver was estimated to be 1.74%, which falls within the 1–2% range estimated to be the lysosomal content of cells, including hepatocytes [44]. Finally, the Western blot analysis showed co-distribution of the late endolysosomal protein markers and Pgp in the first gradient fractions (Figure 1). 

In accordance with LAMP-2 enrichment, the highest Pgp enrichment and recovery were achieved in the first subcellular gradient fraction (Figure 1, Table S4). In contrast, the levels of Pgp detected in fractions containing mitochondrial, canalicular plasma membrane and highest amounts of ER markers were low (Figure 1). 

The canalicular plasma membrane marker ABCB4 was predominantly seen in fractions F9 and F10, whereas only moderate expression of Pgp was observed in these fractions (Figure 1A).

Colocalization of Pgp within LAMP-2 positive vesicles was biochemically analyzed by co-immunoprecipitation analyses in rat liver CLF. To isolate LAMP-2 presenting subcellular vesicles from rat liver, immunoprecipitation was performed with CLF applying two different pulldown methods, centrifugal force or magnetic purification. The immune-purified vesicles were subsequently analyzed for Pgp by Western blotting (Figure S6). Magnetic bead-based isolation next to conventional centrifugal bead pulldown was used to exclude co-isolation of LAMP-2 negative vesicles. Biochemical detection of Pgp in the LAMP-2 immunopurified vesicles is suggestive of Pgp localization in endolysosomes.

**Figure 3:**
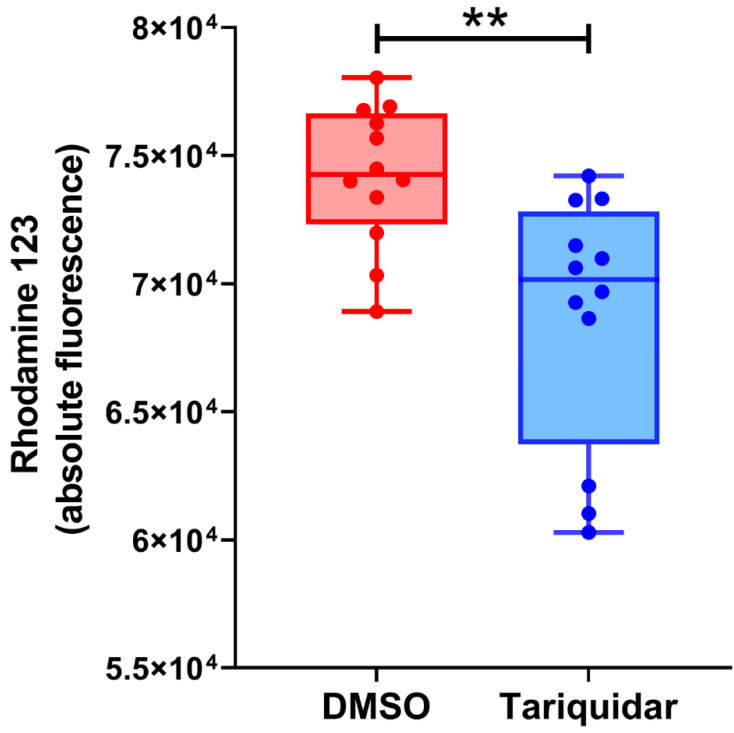
Uptake (or accumulation) of the Pgp substrate rhodamine 123 in the crude endolysosomal fraction of rat liver in the absence or presence of the Pgp inhibitor tariquidar. Tariquidar was used at a concentration of 0.5 µM and rhodamine 123 at 2 µM. Data are shown as boxplots with whiskers from minimum to maximal values; the horizontal line in the boxes represents the median value. In addition, individual data are shown. The experiment was performed 4-times with three replicates per experiment. The significance of the difference is indicated by asterisks (** *p* = 0.0018).

To study whether Pgp in the endolysosomal enriched isolate is functionally active, we used a Rho123 uptake (or accumulation) assay. The fluorescent Rho123 in combination with a Pgp inhibitor is widely used to examine membrane transport by Pgp [45]. As shown in Figure 3, inhibition of Pgp by tariquidar significantly decreased the Rho123 concentration in the CLF of rat liver, which would indicate that inhibition of Pgp activity reduced the uptake of Rho123 into the endolysosomal compartment. This would be in line with the assumption of a topological inversion of Pgp in the endolysosomal system, resulting in the transporter actively pumping agents into these cell organelles [11]. However, although the Rho123 uptake in the tariquidar and vehicle-treated groups was significantly different, most of the individual tariquidar treated samples exhibited a Rho123 uptake that was not different from controls (Figure 3). 

**Figure 4:**
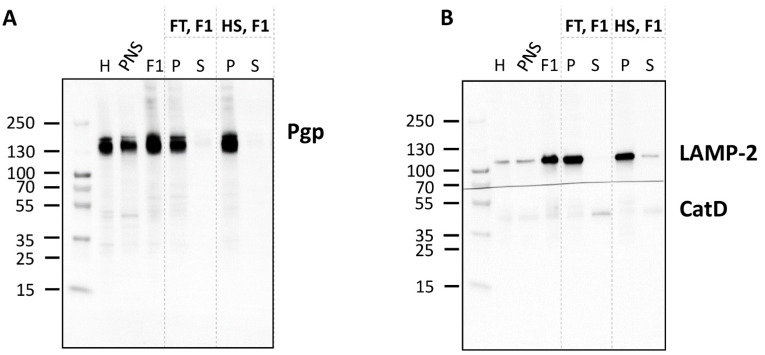
Detection of Pgp in the membrane fraction of F1. The endolysosome-enriched fraction was separated into soluble (luminal) and membrane subcompartments and analyzed by Western blotting for Pgp localization (**A**). The organelle membranes were ruptured applying two different methods in comparison, either repeated freeze–thaw (FT) cycles or a hypoosmotic shock (HS). The pellet (P) enriched in membrane proteins and the supernatant (S) containing soluble luminal proteins were analyzed by Western blotting (5 µg total protein/lane of P and corresponding amount of S). Liver homogenate (H), post-nuclear supernatant (PNS), and fraction 1 (F1) (5 µg total protein each) were analyzed for comparison. (**B**) LAMP-2 was used as a marker protein for the membrane fraction, whereas cathepsin D (CatD) served as a control protein for the soluble endolysosomal protein fraction.

Proteins may be targeted to endolysosomes for degradation in the acidic lumen [46,47]. To determine intraorganelle localization of Pgp in vesicles of the endolysosomal system, organelle membranes were biochemically separated from luminal content. Based on the biochemical analyses of the rat liver subcellular gradient fractions, F1 was selected for the separation of soluble and membrane subcompartments. Therefore, intact endolysosomes were fractured by repeated freeze and thaw cycles or hypoosmotic shock, yielding a supernatant that contains luminal soluble proteins and a membrane pellet after centrifugation. Based on the LAMP-2 protein level, Western blot analysis revealed that gradient purified endolysosomal vesicles were enriched approximately 6-fold in F1 (Figure 4). 

LAMP-2 and CatD were used as marker proteins for the membrane fraction and the soluble luminal fractions, respectively. Indeed, LAMP-2 was primarily detected in the membrane fraction and the luminal enzyme CatD was mainly detected in soluble luminal fractions (Figure 4), validating the separation of membrane and luminal organelle contents.

**Figure 5:**
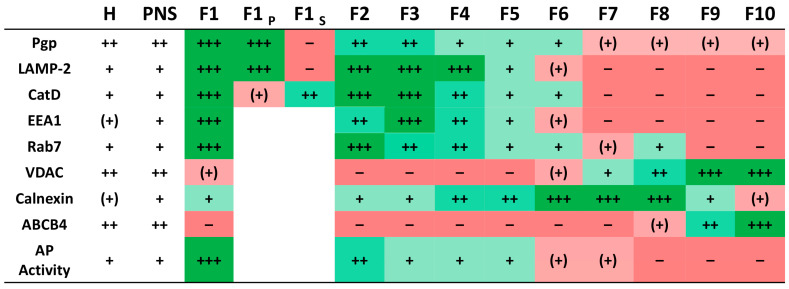
Overview of organelle marker distribution in the gradient fractions. Organelle marker and Pgp distribution in the gradient fractions (F1–10) according to high (+++), medium (++), low (+), or no (−) marker protein content in the respective fraction based on Western blot band intensities or acid phosphatase (AP) activity. Additionally, the Pgp protein level in the membrane enriched pellet (P) isolate and the luminal supernatant (S) resulting from endolysosomal subfractionation of gradient fraction (F) 1 are depicted. The organelle marker distribution is also highlighted by a color code from dark green (high marker content) to dark red (no marker localization). LAMP-2 (lysosomal associated membrane protein 2): endolysosomal marker; integral membrane protein, CatD (cathepsin D): endolysosomal luminal protease; EEA1 (early endosomal antigen 1): early endosomes; Rab7: late endosomal/endolysosomal marker; VDAC (voltage-dependent anion channel): mitochondrial marker; calnexin: ER marker; ABCB4: canalicular plasma membrane marker; AP: acid phosphatase (endolysosomal protease).

Pgp was detected in membrane and not luminal (endolysosomal) subfractions of F1 (Figure 4), indicative of Pgp localization in endolysosomal membranes. However, these data do not allow discriminating Pgp in the limiting membrane of endolysosomes from Pgp in the membrane of intraluminal vesicles (ILVs) in the endolysosomes (see below). Figure 5 summarizes the main biochemical findings of the gradient organelle separation obtained in this study. 

Although based on biochemical data, endolysosomes were enriched in F1, the fraction was not completely free of other vesicles, as for instance indicated by the early endosome marker EEA1. Thus, the biochemical analysis did not allow definite assignment of Pgp to the membrane of specific organelles. Therefore, a series of ultrastructural experiments was performed to (1) analyze the fractions of the gradient organelle separation for purity and vesicle morphology by TEM and (2) examine the in situ localization of Pgp in the rat liver.

### 3.3. Ultrastructural Analyses of the Subcellular Fractions by Negative Staining 

**Figure 6:**
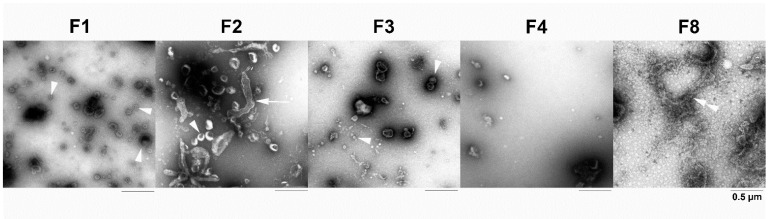
Ultrastructural analyses of the subcellular fractions by negative staining with uranyl acetate. In the negatively stained F1, predominantly round vesicles (arrowheads) with a size of 150 to 250 nm were observed. Endolysosomes typically have an in situ size between 200 nm and >1 µm ([24] see also Section 3.5) but the preparation of F1 likely leads to the shrinking of these organelles. In F2, vesicles (arrowhead) with various diameters from 50 nm to 250 nm and some tubular membranes (arrow) could be recognized. In F3, the different sized vesicular structures were mixed with small protein aggregates, which were diminished in F4. In F8, which was included as a negative control, a tubular network (double arrowhead) was predominantly present. The dark areas in the micrographs are due to higher amounts of uranyl acetate (see Methods).

Structural integrity and homogeneity of vesicles enriched in the different gradient fractions were morphologically characterized by TEM. Only in F1, the vesicle population showed homogeneous morphology and a size of 150 to 250 nm and was essentially free of contaminating organelles of different structures (Figure 6). According to Western blot results, this vesicle population was most likely composed of small endolysosomes, early endosomes (EEA1-positive), and primary lysosomes reacting positively in the AP assay. 

Endolysosomes typically have a size between 200 nm and >1 µm [24]. However, concerning the size of the vesicles, the preparation of F1 may lead to the shrinking of the organelles, so that size alone cannot be used to subtype the vesicles. In F2 and F3, similar vesicles with a diameter of 50–250 nm were observed, but, in contrast to F1, F2 was contaminated with some tubular membranes and small protein aggregates, respectively. The yield rate of vesicles was strongly reduced in F4. In contrast to F1, F8 predominantly contained a tubular network, analogous to mitochondria and ER as detected by Western blot.

### 3.4. Ultrastructural Comparison of Anti-LAMP-2 and Anti-Pgp Immunopurified Vesicles 

**Figure 7:**
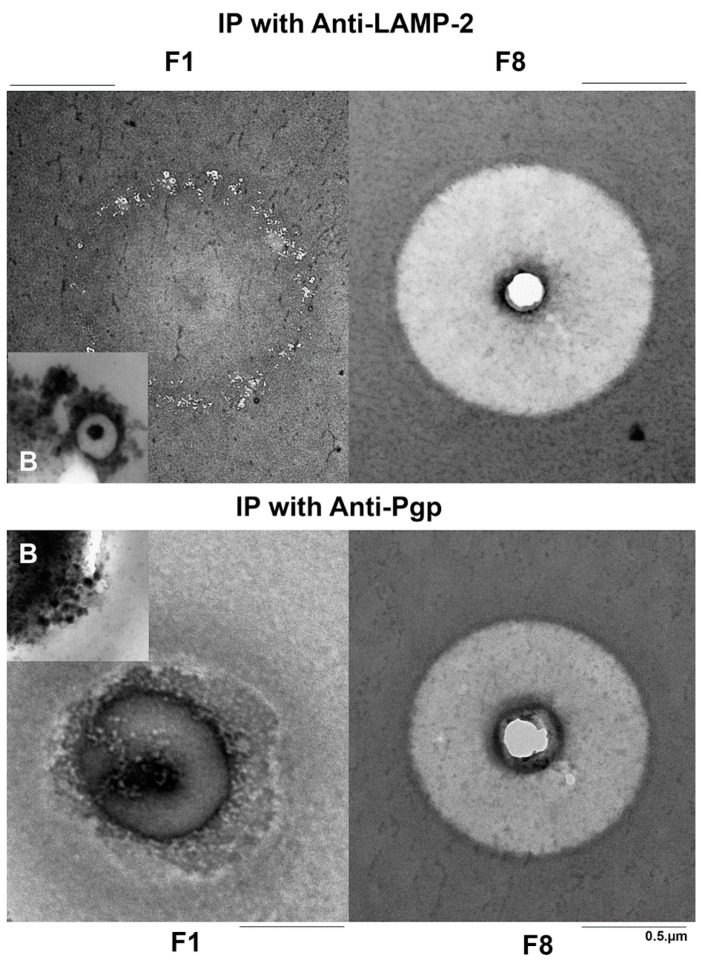
Ultrastructure of anti-LAMP-2 and anti-Pgp immunopurified vesicles. For immunopurification, beads coupled either to an antibody against LAMP-2 or Pgp were used and washed. After negative staining with uranyl acetate, no vesicular structures could be seen in F8, which was included as a negative control. In contrast, in F1 50 nm small LAMP-2 positive vesicles were present on the bead after negative staining. In thin sections of Epon-embedded preparations, multivesicular bodies (MVBs) with a size of about 500 nm were bound to the bead (B) as shown in the inset. When using beads coupled to an antibody against Pgp, only small (about 50 nm) Pgp immunopurified vesicles were isolated and were visible both in the negatively stained and the thin-sectioned beads (inset). In part, these vesicles formed tubular or net-like aggregations.

Morphological conformity of Pgp- and LAMP-2-containing vesicles was evaluated by negative staining of immunoprecipitated fractions F1–F4 and in ultrathin sections of the embedded beads by TEM. By negative contrast, a high amount of about 50 nm small LAMP-2 positive vesicles could be observed in F1, which were absent in F8 (Figure 7). In Epon-embedded preparations, also thin sections of single bound 500 nm multivesicular bodies (MVBs) could be observed, which resembled sectioned endolysosomes (inset in Figure 7). Concerning the Pgp IP, Pgp immunopurified vesicles with a size of about 50 nm were detected in negative contrast as well as in the thin sections (Figure 7); thus, Pgp-positive endolysosomes (as observed with LAMP-2 IP) were not observed. However, by the IP TEM method used, we cannot exclude the presence of Pgp inside endolysosomes if Pgp was predominantly expressed at the membrane of ILVs within the endolysosomes (see Discussion). The small Pgp-positive vesicles observed by IP morphologically resembled transport vesicles or early endosomes. In theory, they could also present ILVs, which range in size from 40 to 100 nm [24]; however, ILVs would have no accessible Pgp epitopes on the cytoplasmatic site that could be precipitated with the C219 antibody. In addition to vesicles, tubular and net-like structures were detected following Pgp IP (Figure 7), which may reflect early endosomes, parts of the trans-Golgi reticulum, or parts of the disrupted canalicular cell membrane, which, however, is unlikely when considering the low/absent expression of ABCB4 in F1 (see above). 

### 3.5. In Situ Localization of Pgp and LAMP-2 in Rat Liver by Pre-embedding Immunogold Electron Microscopy

**Figure 8:**
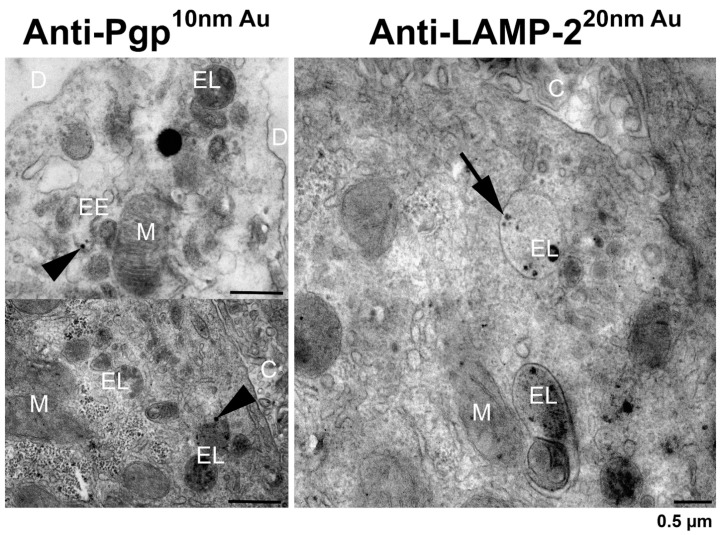
Ultrastructure of the in situ localization of Pgp and LAMP-2 in rat hepatocytes by pre-embedding immunogold labeling. In the hepatocytes, Pgp could be immunolocalized in the early endosomes (arrowhead in EE) near the basolateral plasma membrane as well in one of ten sectioned endolysosomes (arrowhead in EL) near the canalicular plasma membrane (C). Mitochondria (M) are not marked. Space of Disse (D). In comparison, LAMP-2 was only present in the endolysosomes (arrow in EL). Note that—due to the continuous generation of (i) late endosomes by new endocytosed material and (ii) of endolysosomes (or secondary lysosomes) by fusion with primary lysosomes—a differentiation of late endosomes and endolysosomes is not possible by the methods used here, so all these structures were termed endolysosomes (EL). Note that most endolysosomes contain membrane-bound intraluminal vesicles (ILVs); thus, these organelles are also termed multivesicular bodies (MVBs).

**Figure 9:**
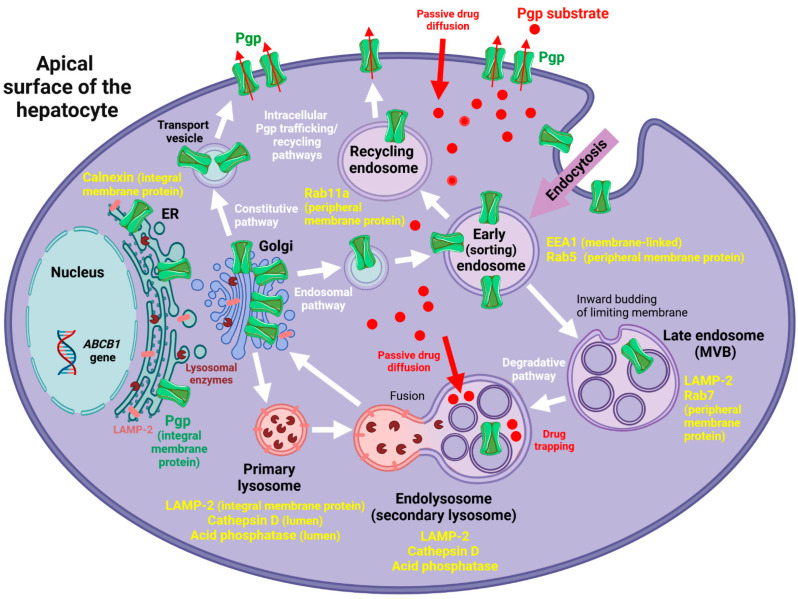
Schematic illustration of synthesis, trafficking, recycling, and degradation of Pgp in a hepatocyte (based on Fu [16] and Löscher and Gericke [6]). Additionally, major steps in the formation of endosomes and lysosomes are shown. Organelle markers are shown in yellow. In addition, passive drug diffusion into the cell and drug trapping of weak-base lipophilic agents (such as doxorubicin) by endolysosomes is illustrated. Please note that the lysosomal enzyme acid phosphatase also occurs in endosomes [25,41], which is not illustrated. At least in part, this enzyme is transported via a constitutive pathway from Golgi via the cell membrane to endosomes and endolysosomes [42,43], which is not illustrated either. A similar constitutive pathway also exists for LAMP-2 [48,49], so transport vesicles and endosomes may contain this membrane protein (not illustrated). The figure was created with BioRender.com (accessed on 7 April 2022).

In prefixed small pieces of the rat liver, the pre-embedding immunogold labeling of LAMP-2 and Pgp was performed to evaluate the Pgp localization in the organelles of the hepatocytes after postfixation with glutaraldehyde and osmium tetroxide as well as staining of the ultrathin sections with uranyl acetate and lead citrate. Pgp could be immunolocalized with an antibody recognizing the cytosolic domain of the transporter predominantly in the early endosomes with a size of 50–100 nm and rarely in a larger endolysosome (in Figure 8 only in one of 10 sectioned endolysosomes). This may support the inward budding from the endosomal-limiting membrane into the endosomal lumen, resulting in Pgp localization within the membrane of ILVs (see Figure 9), which is hardly detectable by the pre-embedding method using a Pgp antibody (C219) to the cytosolic domain of the membrane protein. Morphologically, ILVs were clearly visible in most of the endolysosomes shown in Figure 8. In summary, the in situ TEM data did not demonstrate any robust expression of Pgp at the limiting membrane of endolysosomes. In contrast, and as expected, the immunogold detection of LAMP-2 (also using an antibody to the cytosolic domain of the membrane protein) showed an exclusive localization in the large endolysosomes in the hepatocytes, having a diameter of about 1.2 µm and laying predominantly near the bile canaliculi (Figure 8).

### 3.6. Summary of the Biochemical and Ultrastructural Data 

In Table 1, we summarize the main outcome of the biochemical and ultrastructural experiments on the isolated endolysosome-enriched fraction F1 of rat liver. F1 was used for this comparison because this fraction contained the highest amounts of Pgp, LAMP-2, and AP, and, as shown by TEM, was essentially free of contaminating organelles. Concerning the main question of the present study whether Pgp is functionally expressed in the limiting membrane of lysosomes, no clear evidence for such expression was found. Although the data from membrane fractionation of F1 indicated that, as expected, Pgp is present in the membrane and not luminal subfractions (Figure 4), indicative of Pgp localization in endolysosomal membranes, this does not allow for discrimination between Pgp at the limiting membrane of endolysosomes vs. the membrane of ILVs within the endolysosomes (see Discussion) or the limiting membranes of early endosomes. The Pgp-positive vesicles immunoprecipitated and analyzed by TEM appeared too small to represent endolysosomes but were in line with the smaller size of early endosomes, ILVs, or transport vesicles, which could also contain newly synthesized lysosomal components [50]. However, as described above, for the isolated organelles, the size alone does not allow definite conclusions about the specific nature of the vesicles. 

### 3.7. Electron Microscopy of hCMEC/D3-MDR1-EGFP Cells with vs. without Exposure to Doxorubicin

**Figure 10:**
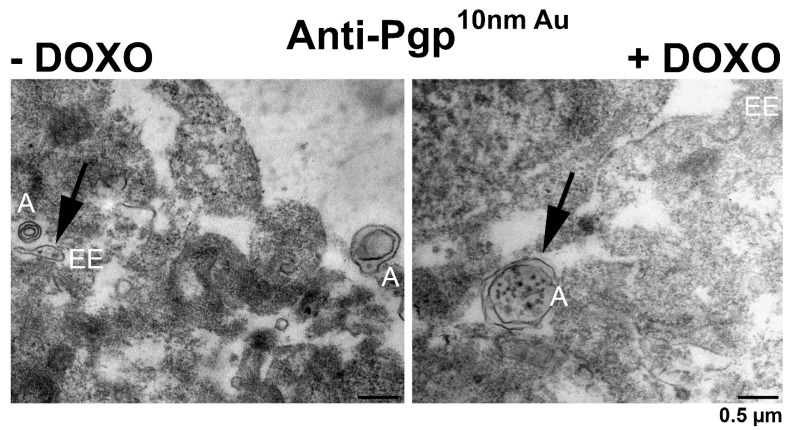
Ultrastructure of the localization of Pgp in immortalized hCMEC/D3-*MDR1*-EGFP cells, in which Pgp-EGFP expression was induced by 1 µg/mL doxycycline. In the left section, cells were sham-exposed, whereas in the right section, cells were exposed for 30 min to the Pgp substrate doxorubicin (DOXO; 10 µM) followed by pre-embedding immunogold labeling. In the sham-exposed cells with small autophagosomes (A), Pgp was predominantly recognized in the early endosomes (EE, arrow). Following doxorubicin exposure, Pgp was immunolocalized in large autophagosomes (A, arrow).

To examine whether Pgp would be expressed at the limiting membrane of endolysosomes in cells exposed to high levels of Pgp substrates, we used an *MDR1*-transduced human BCEC line (hCMEC/D3-*MDR1*-EGFP). In the absence of doxorubicin, Pgp could be immunolocalized at the plasma membrane and in early endosomes (Figure 10) but not in endolysosomes. As in the hepatocytes (Figure 8), LAMP-2 immunogold labeling was predominantly seen in late endosomes/endolysosomes (also termed MVBs) that were characterized by membrane-bound ILVs (not shown). Following exposure to high levels of the Pgp substrate doxorubicin (10 µM for 30 min), Pgp immunogold labeling was predominantly detected in large double-membrane autophagosomes (Figure 10). In LAMP-2 immunogold-labeled sections, LAMP-2 positive autophagolysosomes were observed, in which, however, no Pgp was detected by the mAb used (not shown). Since this antibody (P7965 from Sigma) recognizes an extracellular epitope of Pgp, the lack of detecting Pgp in autophagolysosomes could be due to the rapid degradation of this epitope. 

## 4. Discussion

Lysosomes, or more precisely, the endolysosomal compartment, includes membrane-delimited organelles that are responsible for the degradation of macromolecules, including Pgp, delivered via various cellular pathways comprising endocytosis, phagocytosis, and autophagy [47]. To our knowledge, the group of Des Richardson was the first to propose that Pgp in the lysosomal limiting membrane transports a variety of cytotoxic agents into this organelle; so, lysosomes may act as a “safe house” to prevent cytotoxic effects of Pgp substrates that have surpassed the efflux of the cell membrane-located Pgp [8,11,51,52,53]. The presence of Pgp in lysosomes was demonstrated visually by immunofluorescent staining and confocal microscopy indicating co-localization of Pgp with endolysosomal markers, such as LAMP-2 [8,12,51]. However, confocal microscopy does not allow determining whether Pgp is localized in the limiting lysosomal membrane as suggested by Richardson’s group, or inside the endolysosomal compartment (e.g., in ILVs) as shown previously [16]. In the present study, biochemical methods and ultrastructural analyses by electron microscopy and immunogold labeling were combined to determine the precise localization of Pgp in endolysosomal subcompartments. Furthermore, in previous studies using subcellular fractionation to demonstrate lysosomal Pgp [8,17], contamination of the lysosomal fraction with Pgp-containing plasma membranes was not excluded. Contamination, however, is a general problem of organelle enrichment by density gradient centrifugation and other methods and will influence the final purity of endolysosomes, depending on the specific enrichment protocol used [20,21,22,23]. 

In the present study, contamination of the lysosomal fraction with Pgp-containing plasma membranes was assessed by ABCB4. With the improved subcellular fractionation protocol, ABCB4 expression was negligible in the lysosomal fractions. ABCB4 is almost exclusively localized at the canalicular membrane of hepatocytes and is necessary for the secretion of phospholipids from hepatocytes into bile and for the protection of cell membranes against bile salts [19]. In contrast to Pgp, ABCB4 barely recycles between the plasma membrane and endosomes [38], which makes ABCB4 a suitable plasma membrane marker for subcellular fractionation protocols. In the present study, ABCB4 was mainly detected in gradient fractions F9 and F10, whereas expression of Pgp, which is also expressed at the canalicular membrane, was low in these fractions, which obviously contained plasma membranes that were not previously eliminated from the liver homogenate by low-speed centrifugation. A likely explanation for the different behavior of Pgp and ABCB4 during subcellular fractionation is that—because Pgp recycles between the plasma membrane and early endosomes—it is predominantly located in the early endocytic fractions (F1-4) and less so in F9-10, whereas ABCB4, which hardly recycles [38], is only detectable in F9-10. Another, albeit speculative, explanation for this discrepant expression of ABCB4 and Pgp in fractions F9 and F10 would be that these ABC transporters are expressed in different membrane domains that behave differently during the subcellular fractionation. Pgp is at least partly located in lipid rafts [6,54] whereas ABCB4 located in nonrafts is predominantly involved in the efflux of phospholipids [55]. Further studies are needed to evaluate the apparently different expression of Pgp and ABCB4 in gradient fractions F9 and F10 observed here, but this difference did not affect the main outcome of the present study.

By using an improved subcellular fractionation of native rat liver and TEM of both endolysosome-enriched fractions and intact liver tissue, we demonstrate that Pgp is most likely not localized in the lysosomal limiting membrane in any substantial amount, thus apparently refuting the model suggested by Des Richardson’s group that—due to Pgp expression—lysosomes may act as a drug safe house to prevent cytotoxic drugs from reaching their intracellular targets. Such a mechanism would be not only important in drug resistance of cancer cells but also in the liver, which plays an important role in protecting the organism from potentially toxic chemical insults.

As reviewed by Fu [16] and illustrated in Figure 9, in addition to the plasma membrane, Pgp is localized in many cellular organelles, including ER, Golgi, Golgi transport vesicles, early endosomes, recycling endosomes, late endosomes/MVBs, endolysosomes, and the proteasome. As illustrated in Figure 9, these intracellular localizations link to synthesis (ER), modification (Golgi), trafficking/recycling (Golgi, transport vesicles, and endosomes), and degradation (endolysosomes and proteasome) sites for Pgp [16]. The degradation localization of Pgp in lysosomes appears to be less common within the cells compared to the endosomal localization, which is involved in the constant trafficking/recycling of Pgp between the cellular pool and the plasma membrane [16]. For instance, in MCF-7 cells, 57% of intracellular Pgp-EGFP is co-localized with EEA1 positive early endosomes [15]. However, the endosomal vs. lysosomal localization of Pgp may be affected by the cell type and the physiological state of the cell as indicated by the findings of Katayama et al. [56], who reported a primary subcellular localization of Pgp in the lysosomal degradation pathway of a human colorectal tumor cell line (HCT-15). Furthermore, Victor Ling’s group reported that in multidrug-resistant CH^R^C5 cells, Pgp is detected in the membrane of non-endocytic cytoplasmatic vesicles [57]. For the liver, Kipp et al. [58] have previously reported that ABC transporters such as Pgp cycle between the canalicular membrane of hepatocytes and at least two large intrahepatic ABC transporter pools, one of which corresponds to recycling endosomes. Experiments with EGFP-labeled Pgp in HeLa cells showed that Pgp accumulates in early and late endosomes but not in LAMP-1 positive lysosomes [59], which would be consistent with the present findings. 

However, one possibly important difference between studies reporting functional Pgp in the lysosomal membrane and the present study is the tissue or cell type used. Yamagishi et al. [8] used the human cervical carcinoma-derived (KB31) cell line and the vinblastine-resistant (Pgp-overexpressing) variant KBV1 and reported Pgp-mediated lysosomal drug trapping only in KBV1 cells. Similarly, Liu-Kreyche et al. [17] reported Pgp-mediated lysosomal drug trapping in Pgp-overexpressing tumor cell lines. As pointed out by Szakac and Abele [14], extensive Pgp overexpression will likely overburden trafficking pathways, resulting in mislocalization of Pgp. However, in the Pgp-EGFP overexpressing hCMEC/D3 cells used as a second cell type here, we did not observe any obvious Pgp expression in the limiting membrane of endolysosomes by the TEM techniques used here. 

Importantly, endolysosome enrichment by subcellular fractionation and subsequent biochemical characterization alone may have suggested that LAMP-2 positive organelles (i.e., endolysosomes) localize active Pgp in their limiting membrane. Furthermore, similar to the experiments by Yamagishi et al. [8] in KBV1 cancer cells, transport experiments with Rho123 and a Pgp inhibitor in endolysosome-enriched liver fractions indicated that the endolysosomal Pgp is functionally active, pumping a Pgp substrate into the lumen of the organelles, as also indicated by confocal microscopy. However, as discussed above, the spatial resolution of confocal microscopy as used in the experiments of Yamagishi et al. [8] is too low to allow visualizing whether Pgp is localized in the endolysosomal membrane or in the membrane of ILVs in the lumen of the organelles. As shown in Figure 9, endolysosomes contain membrane-bound ILVs that are formed by inward budding from late endosomes [24]. These ILVs most likely contain Pgp in their membrane. Thus, one explanation for transport data in the present and previous studies in endolysosome-enriched fractions is that transport into ILVs was measured. However, several details argue against the possibility that Pgp actively pumps substrates into ILVs within endolysosomes. First, as shown in Figure 9, as a consequence of the origin of such vesicles by inward budding from the limiting membrane of early endosomes, the topological orientation of Pgp would not allow pumping substrates into ILVs. Second, once sorted to ILVs, Pgp molecules are doomed to destruction by acid hydrolases present in the lysosomal lumen [14,60]. Third, Pgp in ILVs lacks access to ATP, which is needed for active transport [1]. Fourth, the pH in endolysosomes and their ILVs is quite low (pH 4–5), which makes transport by Pgp questionable. Thus, an alternative, more likely explanation for the transport data in endolysosome-enriched fractions is that transport into early endosomes was measured, which contain Pgp in their membrane (Figure 9) and contaminated the endolysosome-enriched fraction. However, as illustrated in Figure 9, the primary function of Pgp in early endosomes is not drug trapping but the sorting of Pgp back to the plasma membrane [16]. 

To our knowledge, TEM has not been used previously to characterize the localization of Pgp in endolysosome-enriched fractions. As shown here, bead-based immunopurification for Pgp and LAMP-2 positive vesicles and TEM indicated that only a subpopulation of small LAMP-2 positive vesicles resembled Pgp-positive vesicles, and this subpopulation was considerably smaller than typical endolysosomes, further refuting the hypothesis that Pgp is present in the limiting membrane of lysosomes in healthy liver tissue. This, however, does not exclude that the endolysosomal limiting membrane expresses other functionally active ABC transporters. In a semi-quantitative proteomic analysis of rat liver lysosome-enriched and lysosome-nonenriched membranes, 734 proteins were significantly enriched in the lysosomal fraction, including nine ABC transporters [61]. Indeed, ABC transporters, such as ABCA2, ABCA3, ABCA5, ABCB6, ABCB9 (or TAPL), and ABCD4, have been classified as lysosomal proteins [14]. Given their membrane orientation (the ATP-binding domains are believed to face the cytoplasm), an exporter function has been predicted for these ABC transporters, implying the extrusion of substrates into lysosomes [14]. However, this simple assumption is not always correct, as demonstrated by the putative reverse transport function of ABCD4, which promotes the transport of cobalamin from the lumen of lysosomes into the cytosol [14]. Interestingly, none of the available proteomic analyses of liver and other tissues or cell lines has identified Pgp (ABCB1) in lysosomal membranes [23,61,62,63]. 

However, this may change if a cell is exposed to high levels of xenobiotics or potentially harmful endogenous compounds, e.g., pro-inflammatory cytokines [6]. For the BBB, we have recently suggested several processes by which Pgp may adapt to high blood levels of xenobiotics to protect the brain parenchyma from intoxication [6]. One of these processes may be intracellular Pgp-mediated drug sequestration in endolysosomes. This assumption was based on a series of studies on BCECs that form the BBB [6,13,37]. In these studies, we used either *MDR1*-EGFP-transduced human and rat BCEC lines (hCMEC/D3 and RBE4) or primary cultured porcine BCECs. As a Pgp substrate, we used either the Pgp substrate eFluxx-ID Gold (EFIG) or doxorubicin. EFIG is a xanthene-based small-molecule dye coupled to acetoxymethyl (AM) ester (EFIG-AM) to allow cell permeability; EFIG-AM has advantages compared with more commonly used Pgp substrates because the hydrophobic, nonfluorescent EFIG-AM readily penetrates the cell membrane, where it is hydrolyzed by intracellular esterases to a hydrophilic fluorescent metabolite (EFIG) that cannot enter intracellular vesicles by passive diffusion [64]. Thus, unless EFIG is actively transported out of the cell or sequestered in intracellular compartments by active transport, the esterase-cleaved dye is trapped inside the cell. Therefore, this feature favors the detection of Pgp-mediated intracellular sequestration in BCECs. In this respect, EFIG differs from more commonly used Pgp substrates, such as weak basic chemotherapeutic agents (e.g., doxorubicin), which can be sequestered in lysosomes by pH trapping in the absence of any active transport by multidrug transporters such as Pgp [10]. In *MDR1*-EGFP-transduced hCMEC/D3 cells, we found that both EGFP-labeled Pgp and EFIG were present in LAMP-2 and LysoTracker-positive organelles [13]. Similar findings were reported for *MDR1*-EGFP-transduced RBE4 cells [37] and primary cultured porcine BCECs [13], excluding that the Pgp/Pgp substrate sequestration was solely due to mislocalization of Pgp in response to extensive expression of Pgp-EGFP. 

In the present study, ultrastructural experiments in hCMEC/D3 cells demonstrated that autophagy in response to doxorubicin exposure induced an extensive localization of Pgp in autophagosomes, whereas expression of Pgp at the limiting membrane of endolysosomes was rarely seen. Autophagosomes ultimately fuse with lysosomes, where the cytosolic cargoes are degraded [65,66,67,68]. Alternatively, fusion of the autophagolysosomes (or autolysosomes) with the Cacoplasma membrane would result in the release of trapped cytosolic components to the cell exterior [69]. We have previously suggested that upon exposure to high levels of Pgp substrates such as EFIG or doxorubicin, lysosomes may fuse with autophagosomes, leading to autolysosomes whose content is released from the cell by outward budding or protrusion of the plasma membrane to form vesicular aggregates (“barrier bodies”) that are attached to the apical cell membrane and are phagocytosed by neutrophils [13]. We proposed that this novel mechanism might contribute to brain protection against potentially toxic xenobiotics, including therapeutically important chemotherapeutic drugs [13]. In cancer cells, it has long been known that autophagy and the fusion of autophagosomes and lysosomes play an important role in drug resistance [65,66,67]. The present TEM data on doxorubicin-exposed hCMEC/D3 cells substantiate that similar mechanisms are operative at the level of the BBB. Although the contents inside the autolysosomal lumen are typically degraded during autophagy [68], a direct fusion of autolysosomes with the plasma membrane and subsequent release of cargo-containing vesicles has been described as a mechanism of unconventional protein secretion [69]. 

Whereas the present experiments do not verify a critical role of Pgp in the limiting membrane of lysosomes for Pgp-mediated lysosomal trapping of Pgp substrates such as doxorubicin, several established lysosomal ABC transporters have been implicated in active drug sequestration [10,14]. The strongest evidence is available for ABCA2 and ABCA3, whereas the potential role of ABCA5 remains to be fully characterized [10,14]. Furthermore, MRP1 (ABCC1) has been implicated in lysosomal drug sequestration [9]. Both doxorubicin and EFIG are not specific substrates of Pgp but are also actively transported by other ABC transporters, including MRPs and breast cancer resistant protein (BCRP; ABCG2) [4,64,70]. Furthermore, doxorubicin is a substrate of the lysosomal ABC transporters ABCA2 and ABCA3 [71]. Based on these findings, one may offer an alternative explanation for our previous finding that—upon exposure of BCECs with EFIG or doxorubicin—both Pgp and EFIG or doxorubicin were co-localized in LAMP-2 and LysoTracker-positive endocytic organelles [13]. This alternative explanation would be that Pgp was present in the lumen of endolysosomes (i.e., in the membranes of ILVs) for degradation, whereas EFIG or doxorubicin was sequestered in these organelles by passive diffusion and/or active transport mediated by ABC transporters other than Pgp. This hypothesis would also explain other data on active drug sequestration in endolysosomes (e.g., [8,17]), because the ABC transport inhibitors used in such studies are not selective for Pgp. 

Since the discovery of lysosomes in the 1950s by Christian de Duve, the rat liver used here has been the principal source of lysosomes for most of the structural and biochemical studies on these organelles [47]. Rather than being uniformly distributed throughout the cell, lysosomes congregate near the biliary pole of the hepatocyte [72,73]. Although lysosomes constitute only a small proportion of hepatocyte volume, they play a key degradative role [74,75]. Apart from their key role in the degradation and recycling of intra- and extracellular material, accumulating evidence indicates that the importance of the lysosome in cell metabolism and organism physiology goes far beyond the simple disposal of cellular garbage [76,77]. As a consequence, lysosomes have emerged as a therapeutic target, not only in the treatment of lysosomal storage disorders but also in more common diseases including inflammatory and autoimmune disorders, neurodegenerative diseases, cancer, and metabolic disorders [77,78,79]. In this respect, the hypothesis of Des Richardson and colleagues that lysosomal Pgp in cancer cells contributes to drug resistance [3,12] is interesting but controversially discussed [12,80]. In the liver, Pgp is located on the canalicular plasma membrane of hepatocytes [16,81]. The present data indicate that, in line with other cell types, Pgp is also present in organelles of the endocytic system; however, the ultrastructural data exclude a predominant Pgp localization in the limiting membrane of endolysosomes under physiological conditions. It remains to be examined whether exposure of liver cells to xenobiotics changes the subcellular expression of Pgp.

To our knowledge, the present study is the first that combines biochemical and ultrastructural methods to examine the endolysosomal localization of Pgp. A combination of biochemical and TEM methods has previously been used to study the endolysosomal localization of other proteins in the liver [44,82]. Without confirmation by TEM, the co-distribution of a protein with an endolysosomal marker does not provide definite proof of its localization in the limiting membrane of endolysosomes. Moreover, microscopic techniques with limited spatial resolution alone, e.g., laser scanning microscopy, may lead to erroneous concepts about the localization and function of Pgp in lysosomes, which is substantiated by the present study. Based on the present data, in endolysosomes Pgp is most likely located in the membrane of ILVs, where it is subject to degradation as illustrated in Figure 9. However, the present study on normal hepatocytes does not exclude that cells exposed to high levels of Pgp substrates or cancer cell lines with abnormal expression of Pgp would express this transporter at the limiting membrane of endolysosomes. Unless this is demonstrated by sufficiently sensitive methods, the debate on the endolysosomal localization and function of Pgp will continue [6,12,14,83], although the present TEM data on Pgp-overexpressing hCMEC/D3 cells seem to disprove that Pgp would be expressed at the limiting membrane of endolysosomes in cells exposed to high levels of Pgp substrates.

In conclusion, by studying the localization–function relationship of Pgp by a combination of biochemical and ultrastructural methods and analysis of Pgp distribution in different subcellular pools that may react to external stress such as increasing concentrations of cytotoxic substrates, we found that Pgp is mainly located in the cell periphery in early endosomes and only rarely in endolysosomes. The Pgp located in the early endosomal pool may react to increasing drug concentrations by redistribution. Additionally, TEM data on Pgp-overexpressing hCMEC/D3 cells exposed to doxorubicin suggest a critical role of autophagy. As a next step, we plan to examine whether the exposure of hepatocytes to high levels of Pgp substrates changes the intracellular localization of Pgp. Furthermore, more recently developed endolysosome purification methods using magnetic, immunomagnetic, or superparamagnetic beads or nanoparticles may be useful to further characterize spatial alterations in lysosomal Pgp [22,23,84]. 

## Figures and Tables

**Table 1:** Summary of the main outcome of the biochemical and ultrastructural characterization of the improved endolysosomal enrichment method. Data were compiled for fraction 1 (F1) of the subcellular fractionation procedure. For details see text.

	Biochemical Data	Ultrastructural Data
Enrichment of endolysosomes in F1?	Yes (indicated by LAMP-2, cathepsin D, and acid phosphatase).	Similar ultrastructure of the negatively stained F1 compared to the anti-LAMP-2 immunopurified vesicles.
Presence of Pgp in endolysosomes?	Possibly; indicated by (1) co-distribution of LAMP-2 (endolysosomal marker) and Pgp in F1 (however, by Western blot, a co-localization of Pgp and LAMP-2 cannot be determined but is not excluded); (2) detection of Pgp in the membrane fraction of F1; (3) Co-IP with LAMP-2 suggests that Pgp is present in the same vesicles but differentiation between localization at the limiting membrane and membranes of intraluminal vesicles (ILVs) is not possible.	Maybe in a subpopulation of small lysosomes (primary lysosomes?) as indicated by the ultrastructure of anti-LAMP-2 and anti-Pgp immunopurified vesicles; however, these small vesicles may also represent transport vesicles, intraluminal vesicles (ILVs) or early endosomes.
Expression of Pgp in the limiting membrane of endolysosomes?	Cannot be determined biochemically, since the membrane fraction of F1 will contain both the limiting membrane of endolysosomes and membranes of ILVs, and, as indicated by EEA1, the limiting membranes of early endosomes; however, the functional data with rhodamine 123 and tariquidar may indicate that Pgp is (weakly) active in organelles such as early endosomes and endolysosomes.	No clear evidence from negative and positive staining of anti-LAMP-2 and anti-Pgp immunopurified vesicles; endolysosomes are only present in the LAMP-2 positive fraction. In situ localization of Pgp and LAMP-2 in rat liver sections indicate that some endolysosomes may express Pgp in their limiting membrane, but—based on the ultrastructural data from F1—this seems to be an exception; in contrast, LAMP-2 is predominantly expressed at the limiting membrane of endolysosomes.
Contamination of F1 with early endosomes (EEA1)?	Yes	Yes, as indicated by the small vesicles in negative stained F1 fraction.
Contamination of F1 with canalicular plasma membranes (ABCB4)?	No	Vesiculated fragments of cell membranes cannot be excluded.
Contamination of F1 with ER (calnexin)?	Yes	A ribosome connected network was not visible in F1.
Contamination of F1 with mitochondria (VDAC)?	No	Mitochondria were not seen.

## Data Availability

Data are available from the corresponding author on request.

## Supplementary Materials

The following supporting information can be downloaded at: https://www.mdpi.com/article/10.3390/cells11091556/s1, Supplementary figures and tables.
