# Supplementary material for: Is P-Glycoprotein Functionally Expressed in the Limiting Membrane of Endolysosomes? A Biochemical and Ultrastructural Study in the Rat Liver
Source: Cells. 2022 May 5;11(9):1556. doi: 10.3390/cells11091556 (PMC9102269; doi:10.3390/cells11091556)
Supplement: Supplementary file 1 [file cells-11-01556-s001.zip › cells-1661909-supplementary.pdf]

Supplementary figures and tables

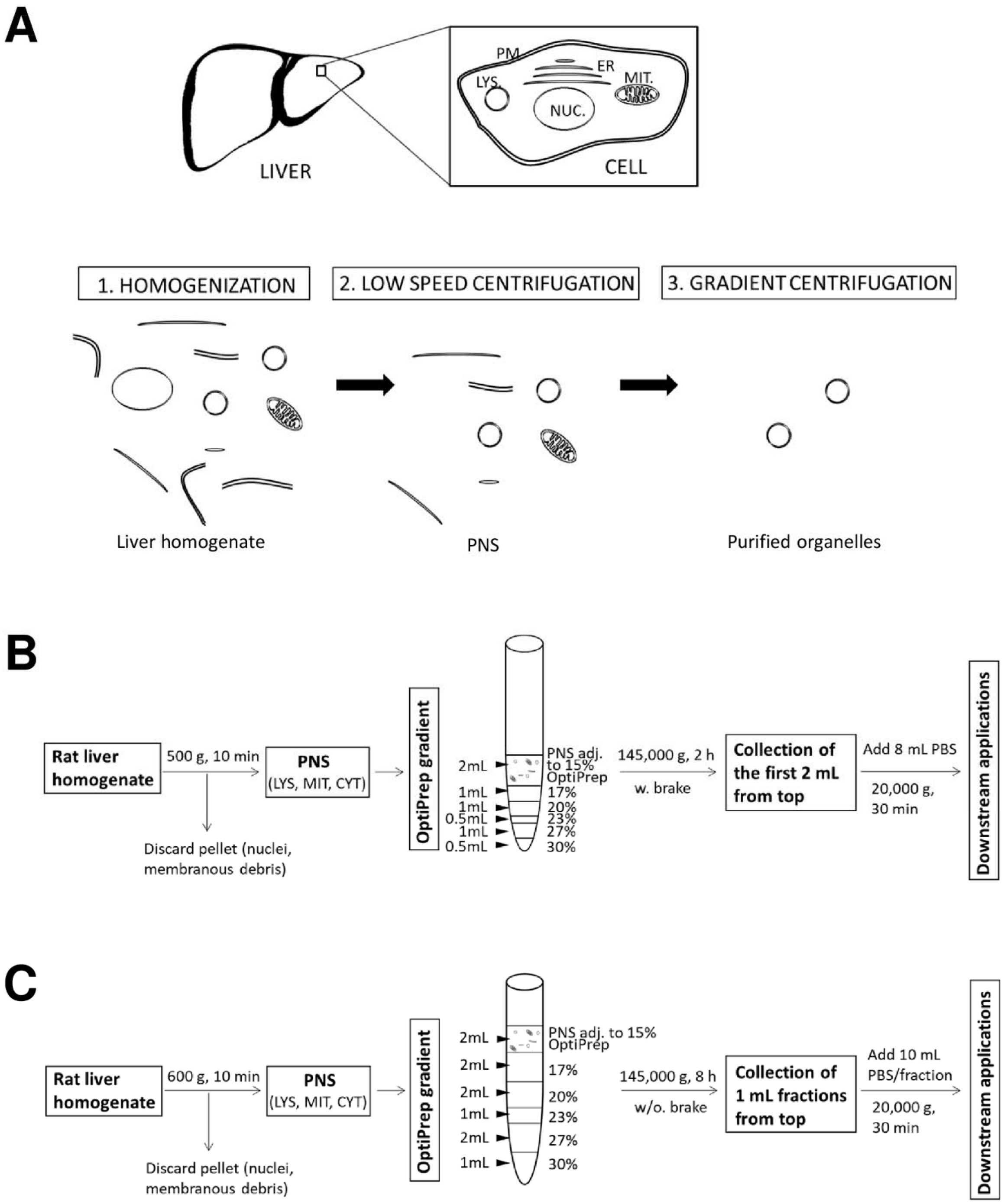

Figure S1.

Simplified scheme of subcellular fractionation procedure for endolysosomal enrichment

from rat liver. (A) Principle of the subcellular fractionation procedure using rat liver tissue. (B) Overview of the basic subcellular fractionation protocol (as described by the vendor of the commercial kit [Thermo Fisher/Pierce]) and (C) the optimized protocol, based on the same kit. Isolated endolysosome-enriched fractions were characterized and Pgp subcellular localization was analyzed by combining biochemical and ultrastructure-imaging approaches. Drawings are not to scale. PM: plasma membrane, LYS: lysosomes, NUC: nucleus, MIT: mitochondria, ER: endoplasmic reticulum, PNS: post-nuclear supernatant, CYT: cytosol.

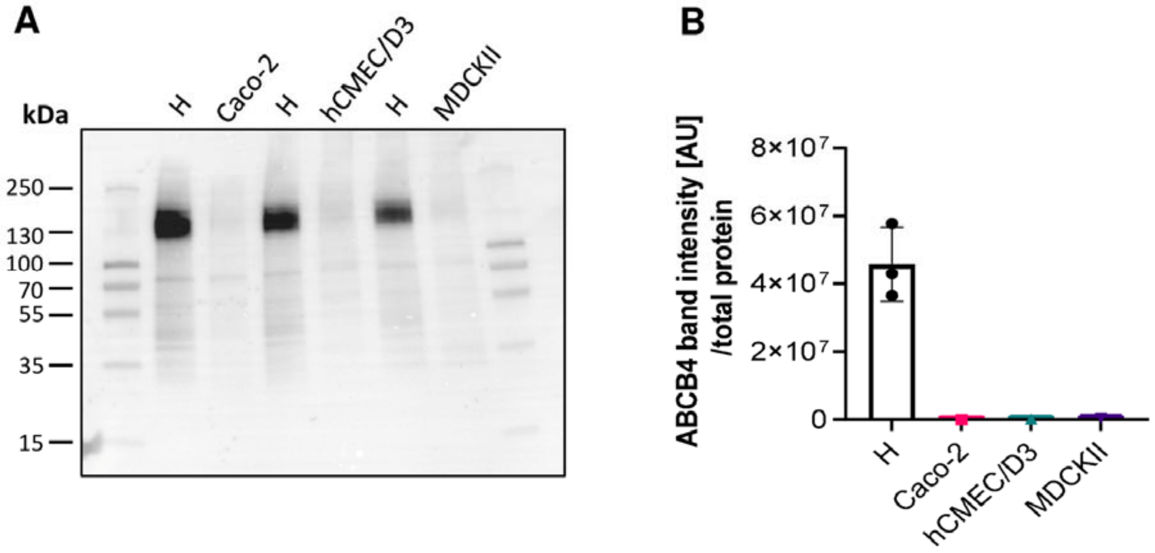

Figure S2.

Validation of ABCB4 antibody specificity in rat liver homogenate by comparison to different non-liver cell lysates. Specific ABCB4 detection by ABCB4 (P2II-26) antibody (Santa Cruz, cat. #sc-58221) in rat liver homogenate (H) was proven by subjecting equal protein amounts (25 µg) of rat liver homogenate and lysates of three different cell lines (Caco-2, hCMEC/D3, MDCKII) to Western blot analysis. (A) A distinct protein band of ~140 kDa for ABCB4 was detected in the liver homogenate but was absent in the cell lysates. (B) ABCB4 bands were normalized to total protein per lane by Stain-Free technology (Bio-Rad Laboratories, Hercules, USA). Data for liver homogenates are shown as mean ± S.D. of three replicates.

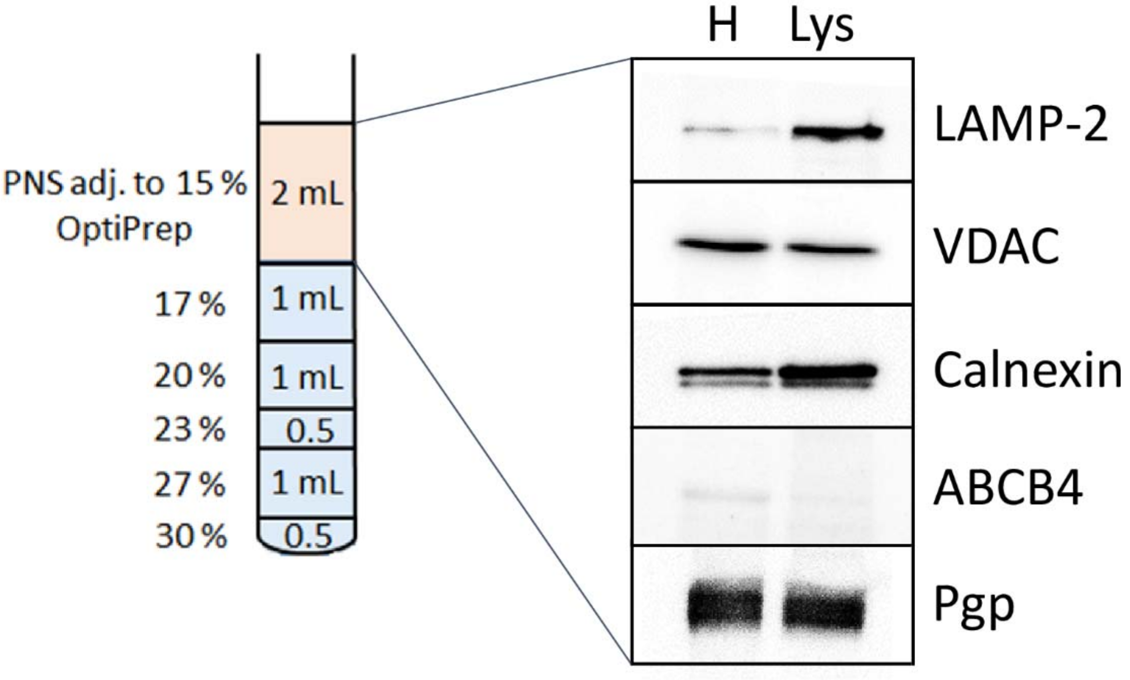

Figure S3.

Biochemical assessment of endolysosome enrichment and organelle purity using the basic protocol for subcellular separation. Rat liver subcellular fractionation was performed following the protocol recommended by the vendor of the commercial kit (Thermo Fisher/Pierce), including low speed centrifugation of rat liver homogenate (H) and density gradient centrifugation (DGU) with the resulting post-nuclear supernatatant (PNS) as described by the scheme depicted. The enrichment and purity of endolysosomes was biochemically assessed by protein organelle marker detection in the potential endolysosome fraction (Lys: upper 2 mL of the gradient after DGU) compared to rat liver homogenate (H) via Western blotting (25 µg of total protein/lane). Compared to H, the endolysosome marker LAMP-2 was enriched in the upper 2 mL of the gradient after DGU alongside with contamination by other organelle markers, i.e., the mitochondrial marker VDAC, the ER marker calnexin, and the apical (canalicular) plasma membrane marker ABCB4.

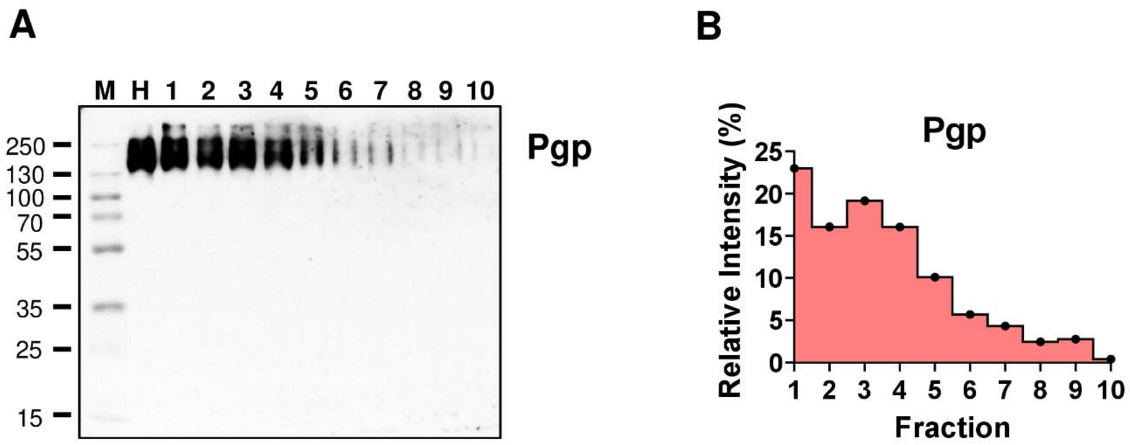

Figure S4.

Pgp distribution in the subcellular fractions determined by applying an antibody that recognizes an extracellular epitope. Subcellular fractionation of fresh rat liver tissue was performed following the optimized protocol and the distribution of Pgp in the gradient fractions was analyzed by Western blotting using a monoclonal Pgp antibody (Sigma, cat. #P7965, clone F4) that recognizes an epitope located in the third extracellular loop of the molecule. (A) Equal protein amounts (5 µg) of liver homogenate (H) and gradient fractions (1-10) were separated by SDS-PAGE and immunoblotted to detect Pgp. (B) Quantification of the Pgp band intensities in the gradient fractions demonstrates that the relative distribution of Pgp detected by the antibody from Sigma is comparable to the distribution obtained by Western blot analysis using the C219 antibody (see Fig. 1). Values are expressed as the percentage of total Pgp levels detected in all fractions combined (set to 100%).

A

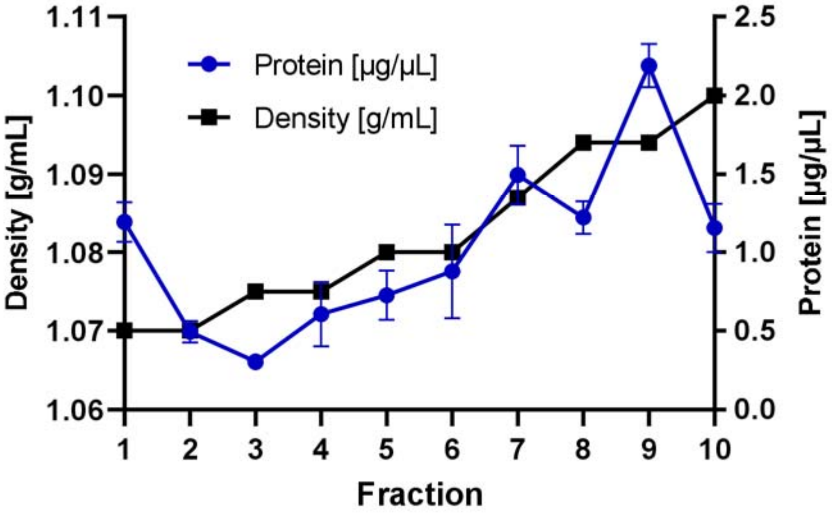

B

Fraction	% OptiPrep	Density [g/mL]
1,2	15	1.070
3,4	17	1.075
5,6	20	1.080
7	23	1.087
8,9	27	1.094
10	30	1.100

Figure S5.

Density of gradient fractions and total protein distribution. (A) Density (black symbols) of OptiPrep gradient layers subdivided in fractions (F) 1-10 and average of total protein content (blue symbols) in the ten fractions collected after gradient fractionation. Data are presented as means ± SEM of seven replicates. (B) OptiPrep concentrations in percentage and corresponding densities in g/mL of the ten 1 mL gradient fractions.

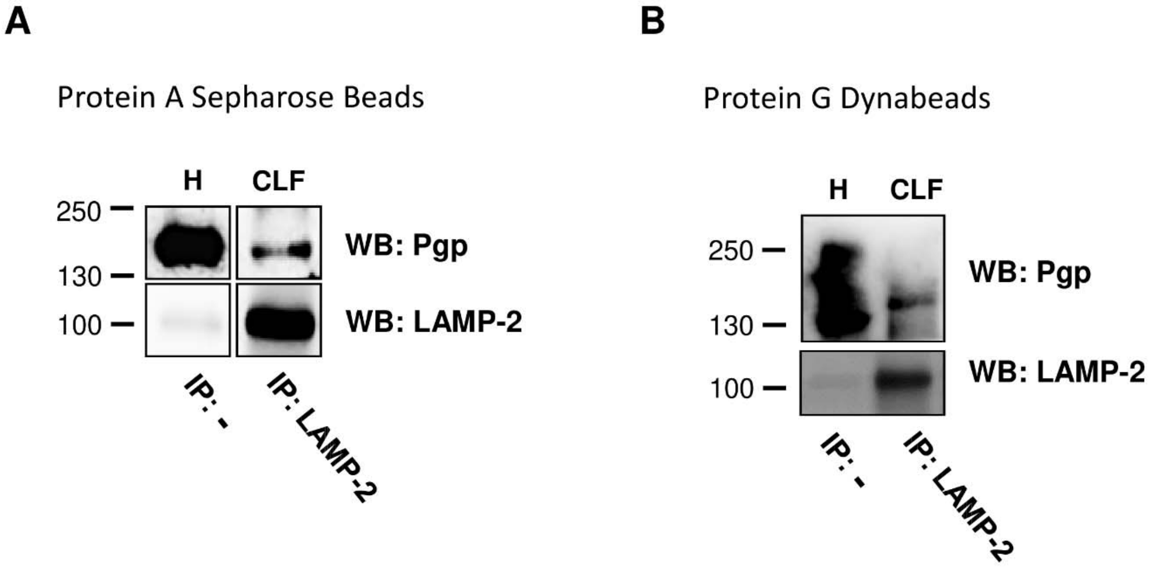

Figure S6.

Codetection of Pgp in LAMP-2 positive vesicles by co-immunoprecipitation. A crude endolysosomal fraction (CLF) was recovered from rat liver homogenate (H) by centrifugation (20,000 x g, 15 min) of the post-nuclear supernatant (PNS). The immunoprecipitation was performed by incubation of the CLF with (A) protein A sepharose (PAS) or (B) protein G Dynabeads coupled to anti-LAMP-2 antibody. After binding to the beads the LAMP-2 positive vesicles were pulled down by (A) centrifugation or (B) by magnetization, washed and analyzed by Western blotting against Pgp and LAMP-2 in comparison to H (15 µg total protein). The precipitated LAMP-2 positive vesicles were also positive for Pgp. WB: Western blot, IP: immunoprecipitation.

Table S1. Comparison of the basic and improved subcellular fractionation protocols. DGU: density gradient fractionation.

	**Basic protocol**	**Improved protocol**
**Low speed centrifugation**	500 x g, 10 min	600 x g, 10 min
**Gradient layer density and volume**	17% (1 mL), 20% (1 mL), 23% (0.5 mL), 27% (1 mL), 30% (0.5 mL)	17% (2 mL), 20% (2 mL), 23% (1 mL), 27% (2 mL), 30% (1 mL)
**DGU speed and time**	145,000 x g, 2 h, w. brake	145,000 x g, 8 h, w./o. brake
**Sampling**	1 fraction top 2 mL	10 fractions of 1 mL

Table S2. Organelle markers used in the present study.

Marker protein/enzyme	Main organelle(s)/subcellular localization(s)
ABCB4<br>(ATP-binding cassette 4)	Apical (canalicular) plasma membrane of hepatocytes
AP<br>(Acid phosphatase)	Luminal endolysosomal hydrolase
Calnexin	Endoplasmic reticulum
CatD<br>(Cathepsin D)	Luminal endolysosomal protease
EEA1<br>(Early endosomal antigen 1)	Early endosomes
LAMP-2<br>(Lysosomal associated antigen 2)	Late endosomes/endolysosomes
Rab7<br>(RAS-related GTP-binding protein 7)	Late endosomes/endolysosomes
VDAC<br>(Voltage dependent anion channel)	Mitochondria

Table S3. Primary antibodies and dilutions used for Western blotting and TEM immunohistochemistry.

Name	Company/Supplier	Catalog Number	Dilution	Host
ABCB4	Santa Cruz	sc-58221	1:500	Mouse
Calnexin	Merck	C4731	1:2000	Rabbit
Cathepsin D	Cell Signaling Technology	69854	1:1000	Rabbit
EEA1	Cell Signaling Technology	2411S	1:1000	Rabbit
LAMP-2	Thermo Fisher Scientific	PA1-655	1:500 - 1:600	Rabbit
Pgp	Thermo Fisher Scientific	MA1-26528 (= C219; used for Western blots)	1:500	Mouse
Pgp	Enzo	ALX-801-002 (= C219; used for TEM immunohistochemistry)	1:500	Mouse
Pgp	Sigma	P7965 (clone F4) (used for Western blots and TEM)	1:500	Mouse
Rab7	Cell Signaling Technology	9367T	1:1000	Rabbit
VDAC	Cell Signaling Technology	4661	1:1000	Rabbit

Table S4. Protein enrichment and recovery of LAMP-2 and Pgp based on Western blot band intensities after gradient fractionation. Fold-enrichment of Pgp and LAMP-2 in the gradient fractions compared to the homogenate (enrichment) and proportion of Pgp and LAMP-2 recovered from the initial amount in liver homogenate (recovery). Highest recovery of LAMP-2 and Pgp was achived in fraction 1. Data are shown as means ± SEM of 7 gradients.

Fraction no.	Enrichment LAMP-2	Recovery LAMP-2 [%]	Enrichment Pgp	Recovery Pgp [%]
**1**	9.61 ±1.89	35.34 ±11.04	1.27 ±0.18	3.34 ±0.90
**2**	8.03 ±0.94	13.17 ±3.83	0.73 ±0.2	1.19 ±0.17
**3**	7.33 ±1.59	8.59 ±3.35	0.65 ±0.24	1.48 ±0.83
**4**	8.12 ±0.66	18.93 ±4.82	0.49 ±0.15	1.09 ±0.42
**5**	4.67 ±1.00	12.68 ±3.26	0.26 ±0.06	0.54 ±0.12
**6**	2.65 ±0.84	6.88 ±1.88	0.22 ±0.05	0.64 ±0.28
**7**	0.68 ±0.17	3.15 ±0.87	0.17 ±0.06	0.67 ±0.33
**8**	0.12 ±0.08	0.42 ±0.24	0.10 ±0.06	0.30 ±0.19
**9**	0.11 ±0.06	0.67 ±0.33	0.08 ±0.05	0.44 ±0.26
**10**	0.07 ±0.04	0.27 ±0.14	0.06 ±0.04	0.19 ±0.14
